# Threshold response to stochasticity in morphogenesis

**DOI:** 10.1371/journal.pone.0210088

**Published:** 2019-01-30

**Authors:** George Courcoubetis, Sammi Ali, Sergey V. Nuzhdin, Paul Marjoram, Stephan Haas

**Affiliations:** 1 Department of Physics and Astronomy, University of Southern California, Los Angeles, California, United States of America; 2 Department of Molecular and Computational Biology, University of Southern California, Los Angeles, California, United States of America; 3 Department of Preventative Medicine, Keck School of Medicine of USC, Los Angeles, California, United States of America; Consiglio Nazionale delle Ricerche, ITALY

## Abstract

During development of biological organisms, multiple complex structures are formed. In many instances, these structures need to exhibit a high degree of order to be functional, although many of their constituents are intrinsically stochastic. Hence, it has been suggested that biological robustness ultimately must rely on complex gene regulatory networks and clean-up mechanisms. Here we explore developmental processes that have evolved inherent robustness against stochasticity. In the context of the Drosophila eye disc, multiple optical units, ommatidia, develop into crystal-like patterns. During the larva-to-pupa stage of metamorphosis, the centers of the ommatidia are specified initially through the diffusion of morphogens, followed by the specification of R8 cells. Establishing the R8 cell is crucial in setting up the geometric, and functional, relationships of cells within an ommatidium and among neighboring ommatidia. Here we study an PDE mathematical model of these spatio-temporal processes in the presence of parametric stochasticity, defining and applying measures that quantify order within the resulting spatial patterns. We observe a universal sigmoidal response to increasing transcriptional noise. Ordered patterns persist up to a threshold noise level in the model parameters. In accordance with prior qualitative observations, as the noise is further increased past a threshold point of no return, these ordered patterns rapidly become disordered. Such robustness in development allows for the accumulation of genetic variation without any observable changes in phenotype. We argue that the observed sigmoidal dependence introduces robustness allowing for sizable amounts of genetic variation and transcriptional noise to be tolerated in natural populations without resulting in phenotype variation.

## Introduction

### Deterministic outcomes from inherently stochastic components

Biological systems are intrinsically noisy but nonetheless produce deterministic outcomes. During development, organisms utilize signaling molecules, i.e. morphogens, to generate a body plan and differentiate cells. With modern experimental techniques, it is possible to measure temporal concentrations of selected morphogens in each cell. The expression of a gene depends on the probabilistic outcomes of several factors, such as molecular binding affinities, processivity, and regulatory sequence interactions. Specifically, genetically identical cells produce morphogen transcripts in asynchronous bursts and in varying quantities [1]. It is therefore essential to investigate how ordered deterministic structures are formed from underlying stochastic components, such as gene expression. In this study, as an approximation to the stochastic nature of morphogenesis, parametric variation is introduced in the partial differential equations of the mathematical model. Earlier studies have addressed robustness of developmental processes, also termed as canalization [2], which remain a subject of great interest today [3–6]. Developmental robustness has been highlighted as a necessary condition that narrows down dramatically the search for plausible models and one that can even “predict key mechanistic and molecular properties of the associated biochemical circuits” [3]. For example, the Turing mechanism has been deemed inapplicable in many instances due to its sensitivity to noise common in developmental processes [6]. In this paper, we analyze the response of a developmental system to noise quantitatively from a statistical physics perspective.

Recent studies in developmental biology have addressed the issue of robustness [7, 8]. For a model to be plausible, there are requirements that the suggested mechanism is robust when parametric noise is introduced. However, in previous works the definition of robustness has mostly been used in a qualitative manner, and the detailed properties of the disordering mechanisms have been left unexplored. In this contribution, novel quantitative measures of robustness are developed and, the value of their application is demonstrated in the analysis of pattern formation in finite-system simulations. These new tools enable the determination of the functional form of the model’s response to stochasticity, leading to a rigorous description of a noise threshold. Specifically, a low-noise regime of robustness is identified in which, even in the presence of stochastic input, there is a deterministic outcome, i.e. structural order. In addition, these new quantitative measures of structural order allow for a precise analysis of the behavior of the system after this threshold is crossed. This disordered regime is relevant to genetic mutations, environmental perturbations and cryptic genetic variation.

### Pattern formation in the Drosophila eye disc is an ideal model system to study the effects of stochasticity on deterministic development

The compound eye, found primarily in insects, consists of an array of repeating visual units. The Drosophila compound eye is made up of approximately 800 unit eyes, known as ommatidia. In wild-type Drosophila, the structure of the ommatidia resembles a near-perfect hexagonal lattice. This highly structured pattern is developed via the delicate coordination of cell signaling, proliferation, movement and apoptosis [9]. Some of these cellular processes are guided through the communication of a few conserved molecules, known as morphogens, resulting in tissue morphogenesis.

While the resulting functional eye emerges in the adult, the role of each cell within repeating ommatidial arrays, and other head structures, are specified during larval development [9]. The larval eye-antenna imaginal disc, hereafter referred to as the eye disc, contains numerous cells that produce various morphogens. The Drosophila eye disc is one of the simplest systems for studying morphogenesis since this tissue develops in an approximately two-dimensional fashion. This is because the eye-antenna disc is derived from an epithelial monolayer [10]. Furthermore, this is an ideal system to model morphogenesis, since cell lineage has a minimal effect on pattern formation, and differentiation relies primarily on cell-to-cell signaling [11]. The underlying developmental mechanisms that guide the formation of the eye disc from larva to adult have been studied extensively, allowing for the generalization and analysis of biologically realistic models.

**Fig 1:**
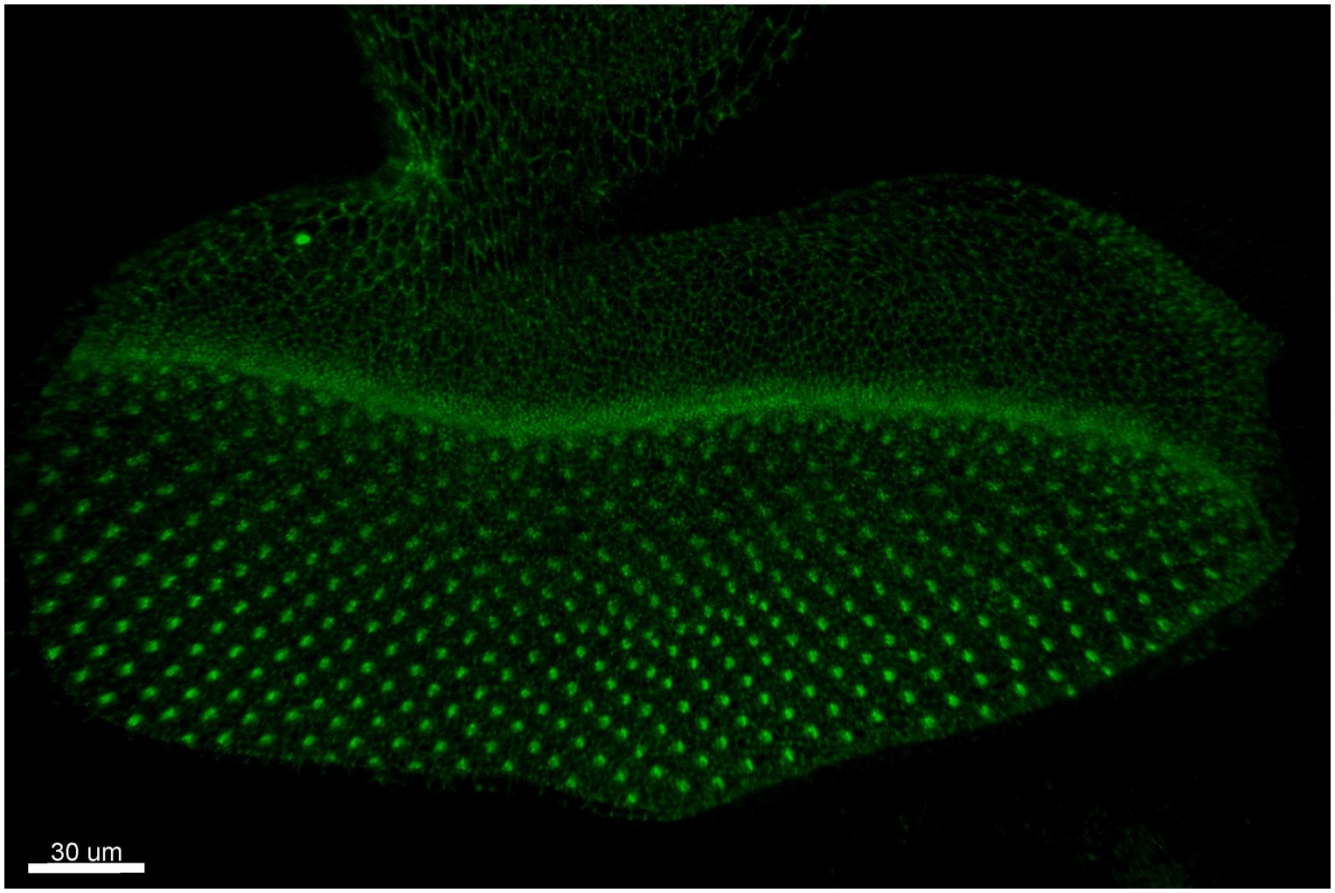
Experimental image of the positions of the R8 cells during morphogenesis. In this fluorescent microscopy image, one can see DE-Cadherin linked with GFP. This labels the top part of each cell shown as a ring. When multiple cells are in close proximity, they appear as one bright point, which in this case point the positions of the R8 cells.

To investigate the stability of the developmental pathways in the Drosophila eye disc, numerical simulations are used, and the results are analyzed from a physics perspective. The spatial organization of the ommatidia begins with the specification of the R8 cells, the future centers of the ommatidia, on the undifferentiated cells of the eye disc, as shown in Fig 1. Mathematical modeling of the Drosophila eye disc pattern-formation mechanism has been the focus of previous investigations, [7, 8] which proposed a model that reproduces the hexagonal lattice pattern of differentiated R8 cells, which is robust enough to be biologically plausible. Using this latest model as a basis, we examine and quantitatively test the robustness of the emerging spatially ordered patterns of differentiated R8 cells when transcriptional noise is introduced. Until today, only qualitative measures of spatial order of the pattern have been applied in this mathematical framework [7]. To this end, we implement several appropriate measures of spatial order, testing the functional relationship of R8 cell pattern order with increasing stochasticity.

During the development of the eye disc, morphogens are produced and emitted by individual cells in quantities that are determined by morphogen inputs from other cells in their vicinity [9]. In addition, the production and diffusion of morphogens is non-uniform, i.e. a given morphogen concentration in a cell does not result in a single production or diffusion rate. Therefore, the highly ordered structure of the ommatidia in the Drosophila eye disc, emerging from such stochastic constituents, requires robustness in the underlying gene regulatory networks. Our study aims to quantify eye disc developmental robustness. We focus on one essential step out of many involved in eye disc development, in which additional robustness is introduced [12]. However, the regulation of R8 cell distances is a crucial developmental step in precisely placing each repeating eye unit, thereby strongly influencing the organism’s overall visual acuity.

### Biological framework of R8 cell specification

Each ommatidium is made of 20 cells, 8 of which are photoreceptors. The specification of the first photoreceptor, R8, guides the specification and orientation of the remainder of the cells within the ommatidium [9]. Thus, the spacing between neighboring R8 cells is pivotal for positioning and refining geometric relationships in resulting ommatidia. Cellular defects that misplace a single ommatidium will influence the position of neighboring ommatidia, thus propagating further flaws in the lattice. If even a single gene involved in ommatidia formation is perturbed, all unit eyes can be affected, since the regulatory logic that generates each ommatidium is repeated using the same set of morphogens and gene networks [9].

Prior to R8 cell specification, the eye disc is composed of tightly-packed undifferentiated cells dividing asynchronously. Differentiation starts with the initiation of the morphogenetic furrow (MF), a physical indentation in the eye disc that sweeps through the tissue to dictate the pace at which ommatidia are specified and positioned. The MF advances anteriorly via the communication of several morphogens, primarily Hedgehog (Hh) and Decapentapegic (Dpp). The former morphogens have been identified as the long range diffusible activators for the production of atonal (ato), the transcription factor that initiates R8 cell differentiation [13, 14]. The MF is initiated in the posterior end of the eye disc and sweeps through the tissue towards the anterior end. As the MF sweeps through the eye disc, the cells posterior to the MF differentiate and commit to their respective role within the ommatidium. Anterior to the MF, the cells are asynchronously proliferating but at the anterior interface of the MF, cells are arrested in G1 to allow synchronous divisions [15].

In the anterior part of the MF, the transcription factor atonal is expressed in intermediate levels in a stripe by all cells. As the MF progresses, the stripe of ato-expressing cells converges to clusters of 12 cells and finally, to a single ato-expressing cell, the R8 cell [9, 16]. The inhibition signals originating from the refining ato-expressing clusters define the template for the refinement of ato-expresing stripes to the subsequent column [14, 16]. Based on the results of [7], which deem the role of Scabrous as a short range diffusible activator, the diffusible inhibitor of stripe refinement has not yet been identified but is related to the epidermal growth factor [14]. Finally, the Notch-Delta pathway is known to be associated with both stripe and cluster refinement [14, 17, 18].

### Process leading to pattern formation in the Drosophila eye disc

In this section, we provide a simplified description of the rational behind the current mathematical model, describing the basic mechanism of pattern formation in the eye disc. The goal is to provide the reader with an intuitive understanding of the foundations of the mathematical model by introducing the minimum amount of elements and interactions. The details of the full model are elaborated in the methods section below.

**Fig 2:**
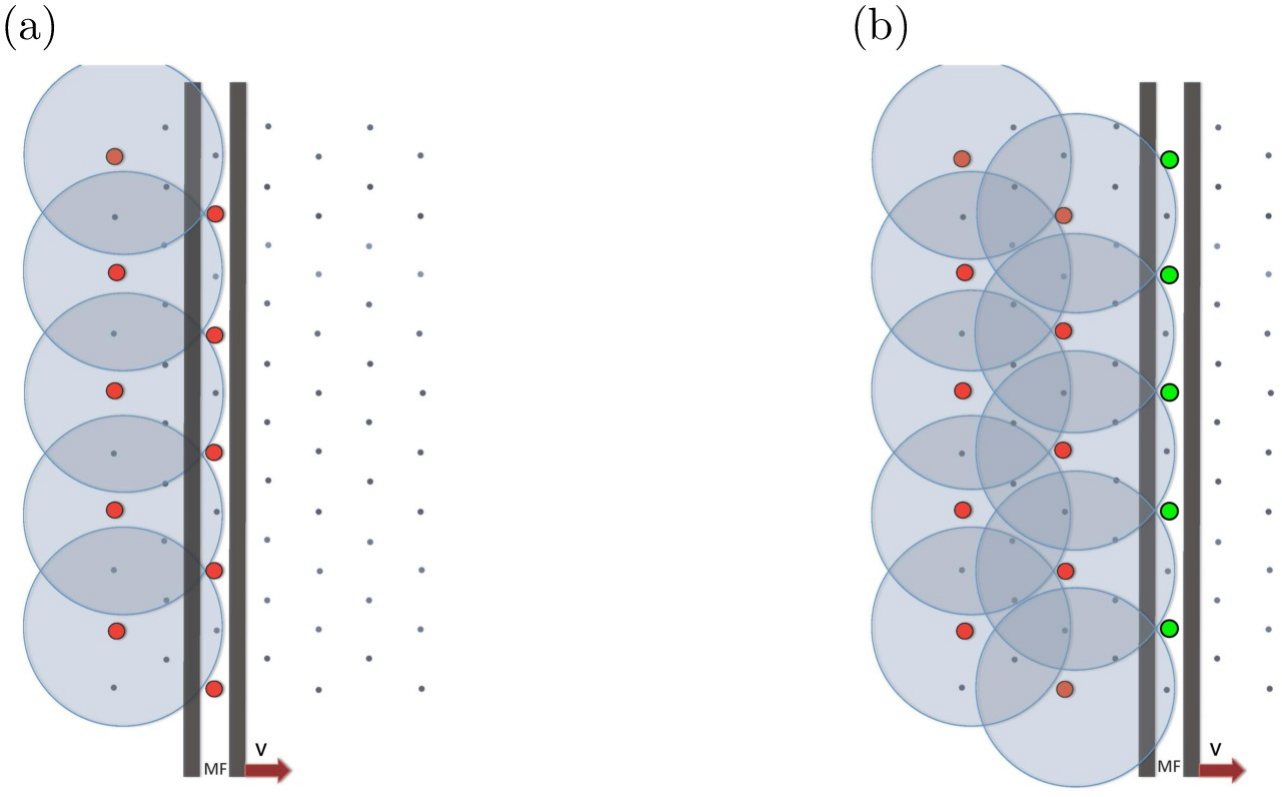
Visualization of the foundations of the R8 cell specification mechanism in the Drosophila eye disc. Simplified illustration of pattern formation mechanism in the Drosophila eye disc as a result of the competition between short-range inhibitor and long-range activator morphogens. Posterior region of the eye disc with an initial row of differentiated precursor cells, denoted by red dots. The morphogenetic furrow (MF), modeled by a plane wave front, moves to the right towards the anterior region. The gray circles represent the boundaries of regions affected by the short-range inhibitor, where the morphogenetic furrow (MF) will not initiate production of *atonal*. Therefore differentiation can only occur in those locations which are not affected by the short-range inhibitor, leading to the hexagonal supper-lattice of differentiated R8 cells, which is experimentally observed. This diagram does not incorporate cluster formation.

The cells of the eye disc are assumed to lie on a hexagonal grid, as appropriate for a hard disk tight packing. After the MF passes, a portion of these cells differentiate to become activated R8 cells, and the remainder will be left undifferentiated. At the end of the process, ato-expressing cells are positioned on a hexagonal lattice. This process is illustrated in Fig 2.

Some morphogens are diffusible, while others are cell-specific and stay within the boundaries of the cell. In this study, *ato* is the only non-diffusing, cell-specific morphogen, while all others can diffuse between cells. The various morphogens either have inhibitory or activating properties. The inhibitors are morphogens that, when present in a cell, decrease the rate of production of another morphogen, and vice-versa for the activators.

The following paragraph defines elements and their interactions in an approximate fashion in order to provide an understanding of the patterning process with minimum complexity. Initially, we will deal with the production of single R8 cells rather than *ato* expressing clusters, which are defined as cells that express *ato* after the MF has passed. There are two main ingredients that make the pattern of R8 cells form. First, there is an inductor (or activator) signal that represents the MF, which causes undifferentiated cells to produce atonal. It can be approximated as a rectangular wave form, with one edge expanding with a constant velocity that eventually moves through the entire hexagonal lattice. Second, there is an inhibition signal that blocks atonal when present above a threshold. When a cell expresses *ato* above a threshold it rapidly, compared to the speed of the MF, produces an inhibitory signal that represses the production rate of *ato* of all cells within its vicinity, depicted by the circular regions in Fig 2. Ultimately, the first cells that do not lie within the inhibitory regions, extending from the most anterior column of ato-expressing cells, will become R8 cells.

As an initial condition, a periodic array of differentiated cells are placed as in the posterior region. The spacing of these cell clusters is chosen such that it will allow for the pattern to propagate. In this simplified setting, the initial configuration is defined by differentiated cells separated evenly in a single row in the posterior-most region. The MF then starts to propagate from posterior to anterior and causes the pattern to propagate as shown in Fig 2.

The actual model that leads to pattern formation is more elaborate. Now that there is a basic understanding of how the pattern propagates, some more intricate aspects can be introduced. In order for the model to be biologically plausible, cell clusters are formed instead of single cells. Even in this illustrative context, as it can be inferred schematically from Fig 2, the propagation of the pattern is very sensitive to the shape of the inhibitory regions. A recent refinement of the model eliminates this flaw by including a short ranged diffusible activator that is emitted rapidly, compared to the speed of the MF, by *ato* expressing cells. In simple terms, this forces the first uninhibited cell to receive the linearly propagating activator signal to create a circular activator region. This activator region defines the cluster size and shape and makes the model robust, preventing the creation of greatly elongated cell clusters, as explained in Ref. [7].

## Methods

### Mathematical formulation

We start with a model of coupled differential equations that describe morphogenetic pattern formation for R8 cell specification on the Drosophila eye disc obtained from [7], an article expanding on the work of [8]. The model relies on the simplifying assumption that the number of cells in the eye disc is fixed [7, 8]. The cells in the eye disc are arranged in a hexagonal grid, treating it as an underlying two-dimensional structure. The lattice constants for the hexagonal lattice are set to 1, and the points of the lattice are defined by the relation $\vec{r}\left(i,j\right)=i\hat{x}+j\left(\frac{1}{2}\hat{x}+\sqrt{\frac{3}{2}}\hat{y}\right)$, where (*i*, *j*) are integers and $\hat{x},\hat{y}$ are the perpendicular unit vectors in the x and y directions respectively. Every cell, due to the hexagonal geometry of the lattice, has six adjacent neighbors. The resulting coupled partial differential equations are of the form:

$$\begin{array}{c} \tau_{a}\frac{da^{i}}{dt}=P_{a}\theta \left(a^{i}-a_{a}\right)-\lambda_{a}a^{i}+G\theta \left(h^{i}-h_{1}\right)\left(1-\theta \left(u^{i}-u_{1}\right)\right)+S\theta \left(s^{i}-s_{1}\right)\left(1-\theta \left(u^{i}-u_{1}\right)\right), \end{array}$$ (1)

$$\begin{array}{c} \tau_{u}\frac{du^{i}}{dt}=P_{u}\theta \left(a^{i}-a_{u}\right)-\lambda_{u}u^{i}+D_{u}\Delta u^{i}, \end{array}$$ (2)

$$\begin{array}{c} \tau_{s}\frac{ds^{i}}{dt}=P_{s}\theta \left(a^{i}-a_{a}\right)-\lambda_{s}s^{i}+D_{s}\Delta s^{i}, \end{array}$$ (3)

$$\begin{array}{c} h^{i}\left(t\right)=\{\begin{array}{c} P_{h}(1-\left(\frac{v\tau_{h}+c_{1}}{2c_{1}}\right)\exp[\frac{-v\tau_{h}+c_{1}}{2D_{h}}\left(y-vt\right)])\;\;y\leq vt\\ P_{h}\left(\frac{-v\tau_{h}+c_{1}}{2c_{1}}\right)\exp[\frac{-v\tau_{h}-c_{1}}{2D_{h}}\left(y-vt\right)]\;y>vt \end{array} \end{array}$$ (4)

**Fig 3:**
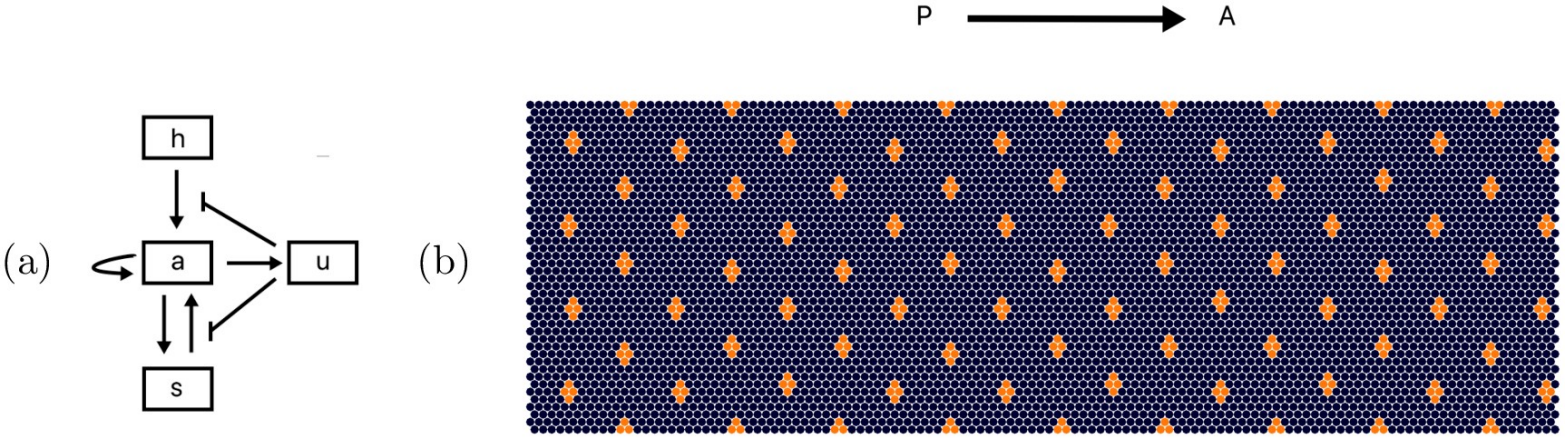
Interactions between morphogens and the simulation of the R8 cell ordered pattern. a) In this diagram we show the interactions defining the regulatory network between the four morphogens in the mathematical model. *h* acts solely as an activator to a, *a* increases production of itself and leads to production of *u* and *s*, *s* activates *a* and lastly, *u* inhibits the effect of *h* and *s*. b) Ordered R8 cell pattern obtained by the mathematical model. The pattern was obtained numerically using the parameters used in [7] (shown in S1 Table).

These represent the spatio-temporal evolution of the morphogen concentrations *a*, *u*, *s*, and *h*, responsible for pattern formation in the drosophila eye disc. The morphogen interactions are summarized in Fig 3(a). Here, the upper case indexes label each cell. The pro-neural transcription factor marking the center of the future ommatidia is *a* (for *atonal*), *u* represents all diffusible inhibitors, *s* denotes short-range diffusible activator (*scabrous*), and *h* (for *hedgehog*) describes the morphogenetic furrow (MF) which activates production of atonal along a propagating wave front. MF propagation is mathematically derived to have this functional form from the underlying morphogen differential equations [7]. The constant *v* is the velocity of the MF, *D*_h_ is the diffusivity, *P*_h_ the production rate, and τ_h_ is the reaction time scale of *hedgehog*.

The mathematical model contains multiple parameters, operators and functions that play important roles in the dynamics of the system. First, the characteristic reaction time scale of each morphogen is given by corresponding τ’s on the left hand side of the equations. In addition, each morphogen’s differential equation contains a term with a −λ coefficient that incorporates the spontaneous decay of the morphogens. This term, combined with τ, determines the mean lifetime of the morphogen via the ratio $\frac{\tau }{\lambda }$. In addition, for the morphogens that diffuse, there are Laplacian operators associated with diffusion, Δ which are discretized at the cell level (see S1 Appendix for exact expression). For cells on the boundary, reflective boundary conditions are used. Note that although only the equations for *u* and *s* have this diffusion term, the differential equations that lead to the functional form of *h* are also based on diffusion. Each Laplacian operator is multiplied by a term *D*, the value of which (when divided by the respective τ) sets the scale for how fast the morphogen diffuses.

The Heaviside functions are essential, as they set the morphogen concentration thresholds that start, accelerate and stop production. They are a simplified version of the more biologically plausible sigmoid functions, and are defined by

$$\begin{array}{c} \theta (x)=1\;x>0,\;\;\theta (x)=0\;x<0. \end{array}$$ (5)

The Heaviside function *θ*(*h*^*i*^ − *h*_1_) in Eq 1 is responsible for initiating production of atonal, *a*, in the target cell with a rate $\frac{G}{\tau_{a}}$, once the concentration of *h* crosses the threshold *h*_1_. This term is multiplied by (1 − *θ*(*u*^*i*^ − *u*_1_)), a function that goes to zero when *u*^*i*^ > *u*^1^. This term introduces the functionality of *u* as a direct inhibitor of atonal in the model. The last term in Eq 1 introduces *s* as an activator of atonal. Furthermore, the first term in Eq 1, *P*_*a*_*θ*(*a*^*i*^ − *a*_*a*_), sets a threshold for atonal at which its production becomes refractive to inhibitory signals. Finally, the two remaining differential equations for the morphogens *u* and *s* in Eqs 2 and 3 have similar functional forms. Here, the Heaviside functions specify the threshold value for atonal in the target cell that is needed for initialization of production.

In this model parameter sets have to be tuned for the experimentally observed ordered pattern to emerge. Furthermore, as the placement of the *ato* expressing cells can vary in cluster size and separation, we use a biologically plausible and stable parameter set, determined by comparison of numeric simulations to experimental imaging data of developing drosophila ommatidia previous stability analyses adopted from [7, 8], shown in S1 Table. More specifically, the decay length scales and time scales for morphogen concentration levels used in the numerics agree with the known experimental data [8]. In Fig 3(b), we show simulation results for this ideal parameter set after the MF has passed the entire cell grid.

The mathematical model is simulated numerically on a hexagonal lattice of 44 vs 120 cells. The size and shape of the simulation grid does not reflect the actual size of the eye disc. The mathematical model evaluated on the 44 vs 120 simulation grid, while using the parameter set shown in S1 Table, results in the differentiation of 85 ommatidia in contrast with the ∼800 ommatidia present in the wild-type eye disc. Moreover, the simulation output contains 20 columns of cells with alternating four to five cells on each column. In contrast, the real eye disc contains 32-34 columns and the number of ommatidia per column varies along the eye disc to provide its elliptical shape [9]. Therefore, the custom size used in the simulations is acceptable to probe how noise propagates between columns while keeping the system size small enough for computational efficiency.

#### Placement of initial clusters, edge effects and the ordered pattern

In order for the pattern to propagate, one must place preexisting clusters of cells with non-zero atonal concentration as an initial condition. Without proper placement, the pattern does not propagate periodically. In this model, a cell is considered to be part of an *ato* expressing cluster when it expresses a concentration of *a* above a threshold value *a*_*a*_. Prior work has not specified a method for determining the initial differentiated cell placement other than trying all possible configurations.

To determine the spacing of the differentiated cells, an isolated differentiated cluster was simulated. After the cell cluster reached a steady state, the shape of the resulting inhibition region was recorded. Then, the spacing between clusters or single cells was determined by requiring an inhibition region that resembles Fig 2. Note that the size of the cluster is determined by a combination of model parameters and was obtained by trials.

In this study, the emerging ordered pattern of R8 cells is not a perfect hexagonal lattice. It is defined as a pattern that retains its structure over long length scales; see S1 Fig for the quantitative definition used. Due to the discreteness of the lattice, for a given radius of inhibition, it is not necessary that a placement of initial clusters exists such that the pattern propagates without slight displacements. In addition, the edges of the simulation grid limit the flawless specification of cell clusters. Specifically, as can be seen in Fig 2, the placement of each *ato* expressing cell cluster is determined by the diffusion of the inhibitor by two *ato* expressing clusters from the previous column. At the edge, only one cluster determines the shape of the inhibitory region leading to a different separation length scale. Finally, even in the biological system, the pattern is not periodic down to single cell positions and a mechanism that requires such fine tuning for a perfect placement of clusters would be unrealistic.

#### Introducing noise

Starting from validated simulations of the noiseless case [7, 8], we are now well positioned to study the effects of parametric disorder on pattern formation. The motivation for this is that even in quasi-identical cells, it is experimentally observed that genes are expressed at appreciably different levels due to stochasticity. Here we investigate how such variations affect the outcome of pattern formation in the Drosophila eye disc. To introduce effects of disorder to the system, one or two parameters of interest are chosen at a time. The reason for this choice is to probe the system response when one of the many components start to fail as often encountered in genetic mutations and other stresses. The analysis for adding equal amount of noise to many parameters and adding variable amounts of noise for a pair of parameters was included for completeness in S2 Fig.

**Fig 4:**
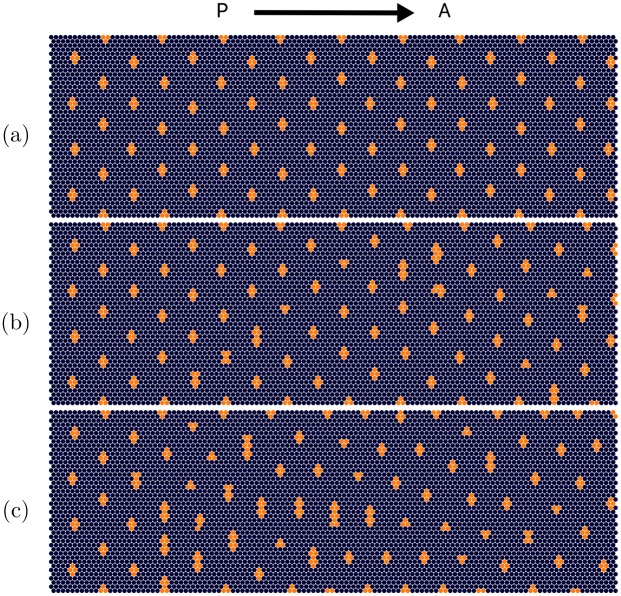
Pattern formation simulation results in the Drosophila eye disc, the morphogenetic furrow moves from left to right. Cluster positions and sizes of *ato* expressing are shown for increasing noise *σ* in the diffusivity of the morphogens *D*_*u*_ and *D*_*s*_. (a)-(c) are the final patterns for *σ*/*μ* = 0, *σ*/*μ* = 30%, and *σ*/*μ* = 40% respectively. Here, *μ* is the mean of the corresponding normal distribution and the value that is used to generate the perfect pattern.

For the chosen model parameters, their value is picked from a normal distribution centered at the mean value that produces the ordered pattern of differentiated R8 clusters in Fig 4(a). For each cell of the system a unique value for the parameter is drawn. They are then kept constant during the time evolution. The width and center of each Gaussian distribution is chosen to be the same for all cells. When more noise is introduced, the width of the distribution is increased accordingly. More specifically, in the numerical data discussed below the widths are tuned from 0% to 60% of the mean. As an example, introducing noise in the production rate of *u* corresponds to picking values from the probability distribution:

$$\begin{array}{c} P\left(P_{u}\right)=\frac{1}{\sqrt{2\pi \sigma^{2}}}\exp\left[-{\left(P_{u}-\mu \right)}^{2}/2\sigma^{2}\right], \end{array}$$ (6)

where *μ* = 〈*P*_*u*_〉. Introducing noise in the diffusivity of the morphogens is a bit more intricate and is explained in S1 Appendix. The effect of introducing noise in the diffusivity of *u* and s is presented in Fig 4. The interpretation of adding noise to the diffusivities corresponds to cell size variation. In this paper, refers to the standard deviation of the normal distribution, as shown for *P*_u_ in Eq 6, and therefore defines the degree of noise. In the unlikely instance that a negative value is drawn from the distribution, the absolute value is considered.

#### Cluster refinement

**Fig 5:**
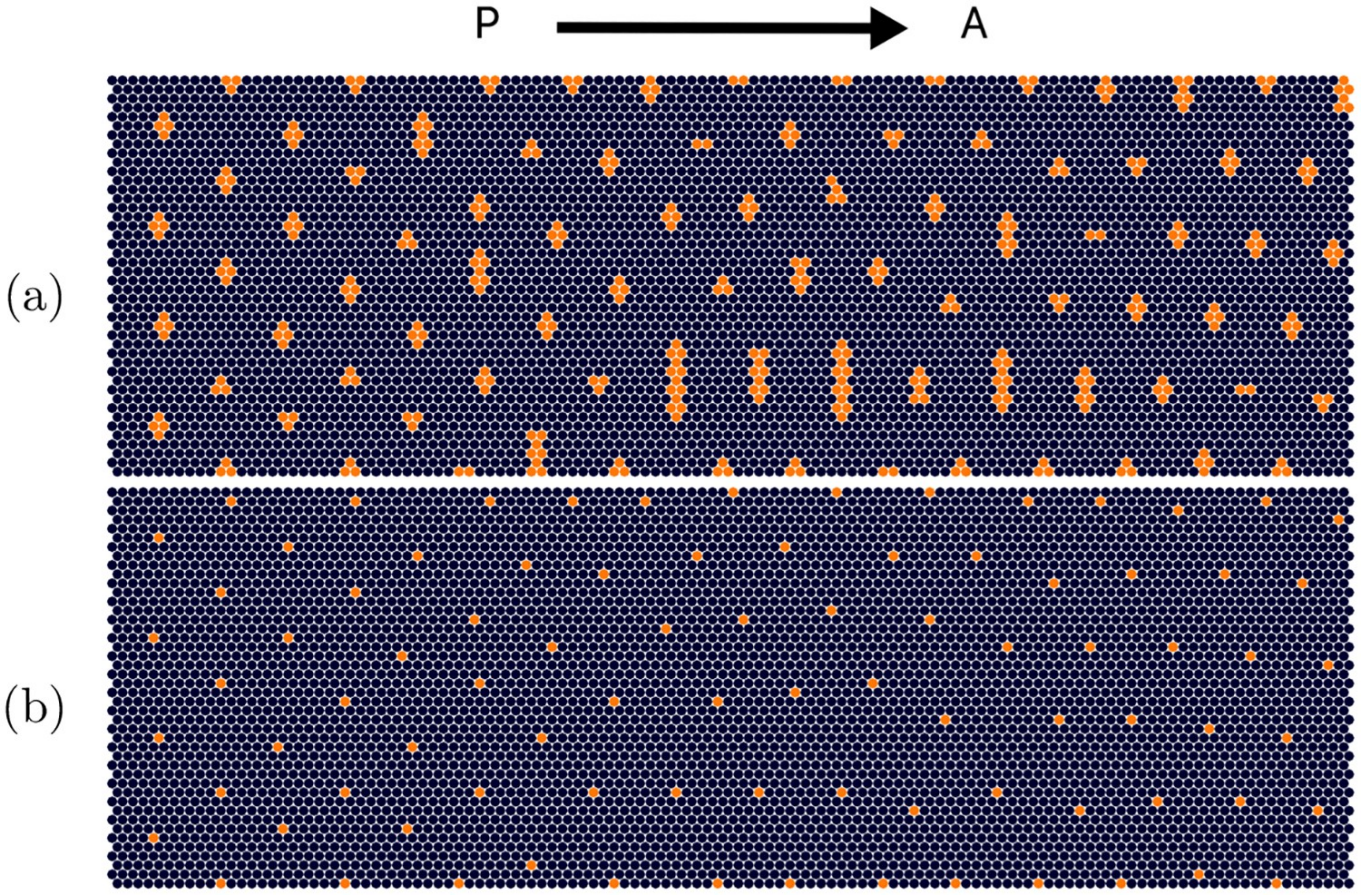
Visualization of the cluster refinement step. The code takes the *ato* expressing cell clusters, as shown in (a), as an input and determines the single R8 cell that will become the center of the future ommatidium, as shown in (b). This example is shown for a noise level of *σ*/*μ* = 40%, applied to the diffusivity of the morphogens *u* and *s*.

Until now, in the parameter regime deemed biologically plausible, the mathematical model in Eqs 1–4 produces patterns containing clusters. However, the actual developmental process includes an extra step that reduces the clusters to single activated R8 cells, which then become the center of the future ommatidia. This process is associated with the Notch-Delta pathway, whereby the cell that produces the most Delta inhibits its nearest neighbors [18]. In order to reproduce this refinement process, a single cell for each cluster is kept as the R8 cell. The cell is chosen to be the most central cell in the most populated row in each cluster, an approximation to the non-trivial pathways and models identified in the literature [7]. This cluster refinement process is illustrated graphically in Fig 5. This extra step, of cluster refinement, has been analytically shown to increase robustness of the pattern [19].

### Numerical evaluation

Numerical forward integration on a 120 × 44 hexagonal lattice with temporal step size 10^−2^ was used to evaluate the spatio-temporal evolution of the coupled differential Eqs 1–4. To obtain sufficient data for statistical analysis, the code was parallelized and run on USC’s high-performance supercomputer cluster center. Each computation was assigned to multiple processors at once, running for around 10 hours on each node.

To obtain reliable error bars, at least 50 realizations of each noise level were simulated. The total eye disc size width was chosen such to eliminate edge effects, allowing sufficient space for clusters to form on the edges. Also, a relatively long eye disk length of 120 lattice sites was used is to allow investigation of error propagation of clusters as a function of position from posterior (P) to anterior (A). The simulations were terminated once the morphogenetic furrow had passed throughout all of the eye disc.

### Quantitative measures of structural order

While previous quantitative studies have concentrated on characterizing the disorder of the R8 point patterns of experiments and creating minimal underlying models [20, 21], in this work we focus on the robustness of the mathematical model and its structural characteristics in the presence of stochasticity. More specifically, in this study the order of the pattern is quantified when varying the noise of parameters that represent regulatory and other processes, distinguishing from order quantification when changing morphological parameters directly [20, 21]. Curiously, while varying morphological parameters directly, a threshold response is also apparent [21]. Furthermore, the order parameter that is developed and applied is distinct from qualitative measures applied before [7, 8], and is more closely related with the measures introduced in quantitative studies [20, 21]. This analysis will provide insight into pathways with which developmental systems either cope with or succumb to stochasticity beyond a threshold, ultimately leading to malformation.

We wish to analyze the emerging point pattern using appropriate measures of structural order, including translational, bond orientation and variance of nearest neighbor distributions. Details of how these measures are designed and applied are discussed in this section.

Traditional measures for determining structural order have been developed in solid state physics [22–25]. However, in the context of the spatio-temporal formation of patterns in biological developmental models there are additional issues to address. Foremost, the degree of structural order in the final pattern is generally not homogeneous in the presence of stochasticity. While, per initial conditions, the posterior point pattern starts off close to an ordered state, it may result in a strongly disordered anterior region at later stages of development, depending on the degree of stochasticity. This type of disorder is correlated between rows because of the manifestly Markovian mechanism that produces the patterns, i.e. the points on a given row are specified based on the geometry of the points in the previous row [26].

This observation renders traditional solid state measures used to evaluate the structure of homogeneous crystals insufficient [23]. Furthermore, the type of correlated disorder exhibited in the point patterns of the eye disc is unlike random thermal displacements found in atoms or infrequent impurities. Instead, such correlated disorder causes Bragg’s law to become inapplicable, since it is based on the assumption that the underlying unperturbed lattice is periodic [22]. Specifically, the model system is far away from the two instances where a corrected Bragg’s law for imperfect lattices could still be applied. The Debye-Waller approximation [27, 28] applies only for uncorrelated deviations from a perfectly periodic lattice, and the available corrections for correlated deviations [29] apply only in the limit of small deviations and short-ranged Gaussian correlations. In order to study the structure of amorphous solids and general highly disordered materials, as in the case of developmental models, it is therefore necessary to resort to the pair distribution function as a local measure of structural order [23].

The definition of the pair distribution function is given by

$$\begin{array}{c} g\left(r\right)=\frac{1}{N}\sum_{j=1}^{N}\delta \left(r-r_{j}\right). \end{array}$$ (7)

In the context of developmental models, this function describes the probability of finding an activated cell at position r, given that another activated cell is located at position *r*_*j*_. The pair distribution when treated as function of the scalar distance, as in the case of isotropic patterns [23], is denoted by *g*_2_(*r*) and is called the radial distribution function.

In the following analysis, we will use a variation of the radial distribution function. Practically, in numerical simulations of eye disk development one can calculate the radial pair distribution function only for small distances. Each simulation of the pattern only contains around 40 activated cells. Moreover, the calculation of *g*_2_(*r*) is limited by the open boundary conditions, because the application of periodic boundary conditions to a single realization and the combination of multiple realizations is unphysical. Therefore, here we focus on a variation of the radial distribution function, that includes only nearest neighbors. The nearest neighbors were identified via the Voronoi tessellation method [30].

Furthermore, we analyze a scalar measure of the spatial order of the patterns, the translational order parameter defined as

$$\begin{array}{c} T=\frac{\int_{0}^{\eta_{c}}\left|1-g_{2}\left(r\right)\right|dr}{\eta_{c}}, \end{array}$$ (8)

where η_c_ is a cutoff limited by the simulation size [24]. This order parameter can be interpreted as the Kolmogorov probability distance [31] between the radial distribution function of the target pattern and the Poisson random point process. (The radial distribution function of a Poisson random point process is the uniform distribution, which in the normalization used in Eq 8 is equal to 1). The translational order parameter, *T*, is a general order metric used to describe systems independently of the underlying crystal structure. In this setting, we examine how fast the pattern deteriorates compared to the perfect pattern, so we replace the uniform probability distribution with that of the perfect pattern. In addition, since we only considered the radial distribution of nearest neighbors, the cutoff _c_ in the integration in Eq 8 was replaced with the furthest nearest neighbor distance.

Finally, we study the bond angle order parameter. Contrary to the translational measure, this order parameter evaluates the spatial orientation of vectors connecting the nearest neighbors of all points. It is defined by

$$\begin{array}{c} q_{6}=\left|\frac{1}{N}\sum_{j=1}^{N}\sum_{k=1}^{N_{n}}\exp\left(6i\theta_{jk}\right)\right|, \end{array}$$ (9)

[25], taking a value of 1 for a perfect hexagonal point pattern and 0 for a completely random pattern. Here *N*_*n*_ is the total number of nearest neighbors in the point pattern, the j’s sum over all activated cells, and the k’s sum over all nearest neighbors of a given reference point. Lastly, *θ*_*jk*_ is the angle of the vector connecting each point with its nearest neighbor with respect to a fixed axis.

The discussion in this section connects and motivates the order measures used in this article with the tools available in the literature. It is important to note that equations Eqs 7 and 8 apply to point patterns in continuous space. In this article, the point patterns are defined by positions of the activated R8 cells which are constrained to lie on a hexagonal lattice. This means that the argument of the pair distribution function becomes discrete. Thus, we are going to consider discrete probability functions. This leads to the scalar pair distribution function to be defined as:

$$\begin{array}{c} g_{2}\left(r_{i}\right)=\frac{1}{N}\sum_{j=1}^{N_{bins}}\delta_{ij}n_{j}, \end{array}$$ (10)

where *N*_*bins*_ is the number of bins used, *n*_*j*_ the frequency of activated cells at the jth bin and *N* is the total number of R8 cells. Similarly, the translational order parameter is defined as:

$$\begin{array}{c} T=\sum_{j=1}^{N_{bins}}\left|\frac{\Delta x_{j}}{\eta_{c}}-g_{2}\left(r_{j}\right)\right|. \end{array}$$ (11)

#### Probability distribution functions of nearest-neighbor distances and angles

To quantitatively determine the degree of disorder, a post-processing code is used to record the activated R8 cell positions in the final patterns and generate the probability distributions of nearest-neighbor angles and distances. Firstly, the R8 clusters are refined to single R8 cells by the cluster refinement method explained above. For every R8 cell, the nearest neighbors are identified via the Voronoi tesselation [30].

**Fig 6:**
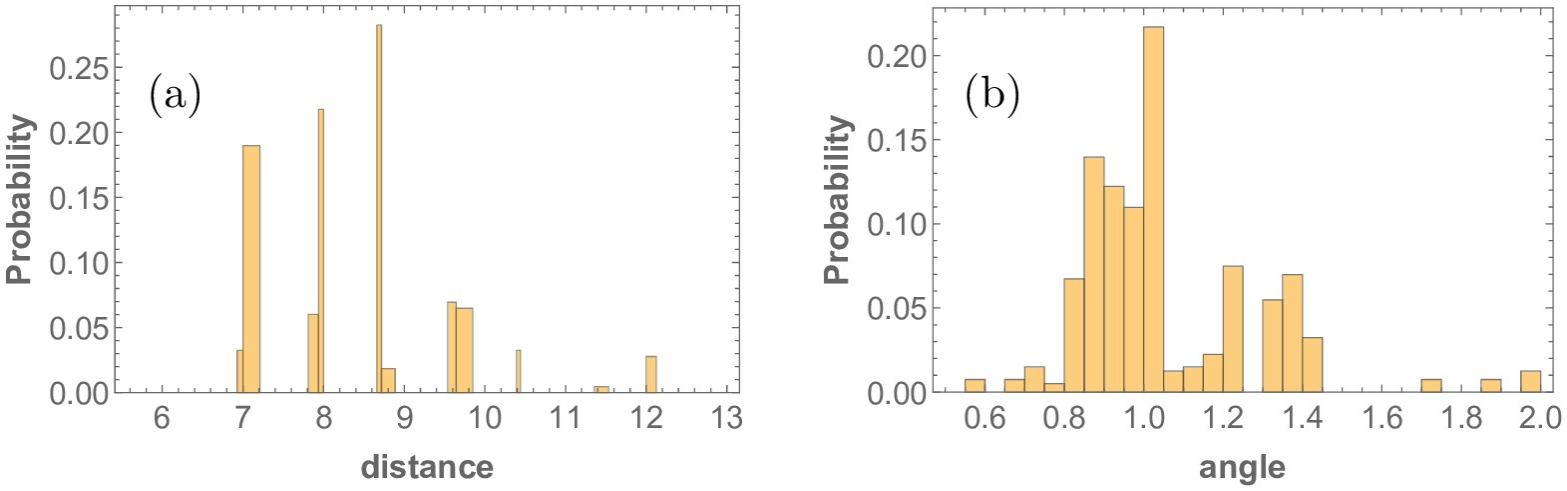
Nearest neighbor (a) distance and (b) angle probability distributions of R8 cell point pattern in the Drosophila eye disc in the absence of stochasticity. Distances are naturally binned by the available sites on the underlying hexagonal lattice, whereas angular positions are collected in bins of 0.05 radians.

Next, nearest-neighbor distances and angles of each R8 cell are calculated, and a filter is applied to correct for boundary effects for the nearest neighbor angles. Combining this information for all random realizations, the probability distribution functions of nearest-neighbor distances and angles are determined. Since the cells in the simulation are positioned on a hexagonal lattice, the nearest-neighbor distances and angles take discrete values. As a consequence, the probability distributions are also discrete. They are shown in Fig 6 for the ordered case. These types of probability distribution functions are used to quantify the order of the R8 cell point patterns by calculating the variance and the distance between probability distributions. The same approach can be taken when interpreting experimental data obtained from imaging.

Even in the zero noise case, the histograms contain multiple peaks in Fig 6. The histogram peaks for the nearest neighbor distances are a consequence of three effects. First, there are two length scales associated with the nearest neighbor distances in the pattern, one for the x-axis and one for the y-axis. Even for a perfect hexagonal lattice there are two lattice constants, one for each dimension. Second, cells in the edges of the simulation grid are placed in a different separation than the cells in the bulk. The two former points result to four peaks. Third, by the definition of nearest neighbor dictated by the Voronoi tessellation, the edge cells along the y-direction boundaries are nearest neighbors of each other creating a fifth peak. The very short peaks that remain in the rightmost side of Fig 6(a) are a consequence of edge effects and the Voronoi tessellation in the x-direction boundary. Finally, slight blurring of each peak is a consequence of clusters forming one or two cells away than their counterparts.

#### Variance as a measure of order

The nearest-neighbor angles and distances can be thought of as samples of an underlying distribution. If this distribution has non-vanishing higher-order moments, it is not trivial to produce a measure of disorder. However, in the simple case where the distribution can be approximated by a Gaussian, its variance, can be used as a reliable measure of the spread of the distribution. The variance used in this context is the sample variance of the nearest neighbor distances and angles. It is calculated from N values, *x*_*i*_, using

$$\begin{array}{c} s^{2}=\frac{1}{N-1}\sum_{i=1}^{N}{\left(\bar{x}-x_{i}\right)}^{2}, \end{array}$$ (12)

where $\bar{x}$ is the sample mean.

#### Probability distribution distance as a measure of order: Fidelity and Kolmogorov distance

Here we introduce two new and useful measures of order which best address the needs of this study: fidelity and Kolmogorov distance. Both of these rely on the concept of distance between probability distributions, and are inspired by the translational order parameter *T* defined in Eq 8. The concept of probability distance applied as an order measure is, to the best of our knowledge, novel. The most novel adjustment is to apply the measure of probability distance not only on the nearest neighbor distances distributions but also the nearest neighbor angles distributions. Note that given any set of probability distributions that include an ordered case, of any dimension and type of variable, the order measures introduced here can be applied to quantify deviation from the ordered state. To define these measures of order, we use the zero noise case probability distribution as a reference and calculate its distance from each of the noisy probability distributions. This choice is intuitive, since we are addressing the question of how disordered is the pattern relative to the perfect pattern. The results did not depend strongly on the reference distribution, see S3 Fig for the results when using Eq 11 where the reference distribution is the uniform distribution.

The fidelity of two discrete probability distributions *p*(*x*_*i*_) and *q*(*x*_*i*_) is defined as

$$\begin{array}{c} F\left(p\left(x_{i}\right),q\left(x_{i}\right)\right)=\sum_{k}\sqrt{p\left(x_{k}\right)q\left(x_{k}\right)}, \end{array}$$ (13)

where the sum runs over all the bins of the discrete distributions [31]. The fidelity between two distribution falls into the range 0 ≤ *F*(*p*(*x*_*i*_), *q*(*x*_*i*_)) ≤ 1, where 1 is attained only when *p*(*x*_*i*_) = *q*(*x*_*i*_) ∀ *x*_*i*_. The Kolmogorov distance, in this context, is defined as

$$\begin{array}{c} D\left(p\left(x_{i}\right),q\left(x_{i}\right)\right)=\frac{1}{2}\sum_{k}\left|p\left(x_{k}\right)-q\left(x_{k}\right)\right|, \end{array}$$ (14)

where the sum again extends over all the bins of the discrete distributions [31]. Akin to the fidelity, the Kolmogorov distance is bounded by zero and one. This measure represents the maximum deviation of the two probability distribution functions given that a collection of events occur. In order for the Kolmogorov distance to match the functional form of the fidelity, we will consider 1 − *D*(*p*(*x*_*i*_), *q*(*x*_*i*_)) instead. This quantity is 1 when the two probabilities are the same and 0 when they do not have a single common non-zero element.

## Results and interpretation

### Threshold response

**Fig 7:**
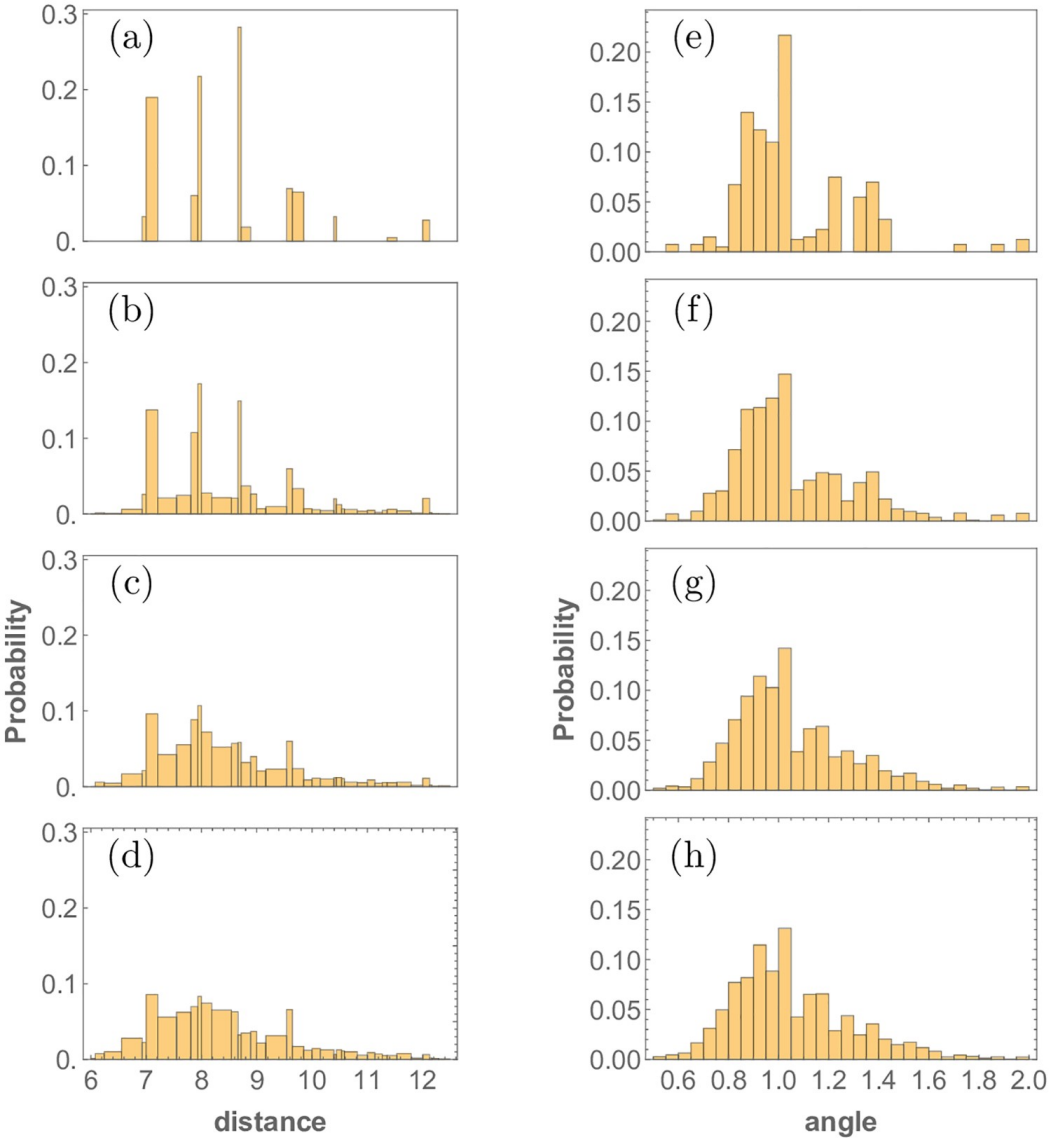
Nearest neighbor distance (left panels) and angle (right panels) probability distributions generated from the point-pattern in the Drosophila eye disc R8 cell specification for increasing noise levels. Stochasticity was introduced in the differential equations by drawing the value of the diffusion coefficients *D*_*u*_ and *D*_*s*_ from a normal distribution with mean *μ* and standard deviation *σ*. The nearest neighbor distance histograms in (a) to (d) and nearest neighbor angle histograms in (e) to (h) correspond Gaussian stochasticity with standard deviations *σ*/*μ* = 0%, 30%, 40%, 60%. While the first two histograms are almost identical, as noise in the diffusivity of the morphogens increases, the peaks get smeared out and the histograms appear wider.

To quantify disorder in the emerging activated R8 cell patterns, the nearest neighbor distance and angle probably distribution functions were computed for various noise levels in the diffusion coefficients *D*_*u*_, *D*_*s*_. (Later we will discuss the effect of stochasticity on other model parameters). Their histograms are shown in Fig 7. The binning used for the distance histograms reflects the discreteness of the underlying hexagonal lattice on which the cell centers are placed. For the distance diagrams, all possible nearest neighbor distances on the hexagonal lattice are used as bins. For the angle histograms, since there are many more possibilities, a constant bin size of 0.05 radians was chosen in order to appropriately resolve the distribution.

**Fig 8:**
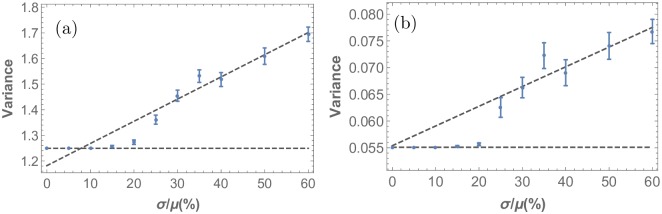
Disorder in the R8 cell point pattern of the developing Drosophila eye disc as a function of stochasticity. Here, the variance of the distributions of (a) nearest neighbor distances and (b) nearest neighbor angles is used to quantify the amount of disorder. As in the previous figure, stochasticity is introduced in the diffusivity of the morphogens *D*_*u*_ and *D*_*s*_, and the x-axis represents the noise level in terms of the standard deviation of the underlying Gaussian distribution from which this parameter is drawn. As the noise level in the simulation is increased beyond a threshold, the variance grows.

The angle histograms, unlike the distance histograms, are skewed towards larger angles. This asymmetry makes the analysis of the distributions more complicated, as in this case the variance, paints an incomplete picture. As discussed below, the probability distance order measure eliminates this issue. The angle histograms are skewed as a direct consequence of the cluster refinement process. As seen in the plots of atonal patterns (Fig 4), the onset of disorder is signaled by elongated clusters of activated R8 cells. Since these elongated clusters are brought down to a single cell in the cluster refinement step, and they cast large inhibition radii, the number of nearest neighbors found for the elongated clusters is less than the six found in the perfect pattern. A lesser number of neighbors trivially leads to higher angles in this case. Both histograms, distances and angles, start out sparse for the case without any stochasticity in *D*_*u*_, with empty bins between peaks, indicative of the highly ordered repeating lattice structure produced by the simulations in this case. As stochasticity increases, the resulting point patterns become aperiodic, leading to denser histograms. Furthermore, the histograms become wider with increasing stochasticity. This is captured in the variance, shown in Fig 8.

The variance plots in Fig 8 illustrate the generic nature of robust pattern formation in stochastic developmental models. They show that for small levels of stochasticity (here *σ*/*μ* < *σ*_*c*_/*μ* = 20%) the ordered pattern remains basically unaffected. However, beyond this threshold the variance starts increasing linearly, both in the angle and distance histograms. This threshold response is a central finding of this study. As discussed below, this is a universal phenomenon, largely independent of which model parameters experience stochasticity and which measures of order are used to assess the system response. It implies that these stochastic models capture biological resilience against stochastic variation up to a certain point, resulting in a regime of deterministic outcomes in spite of stochastic input.

**Fig 9:**
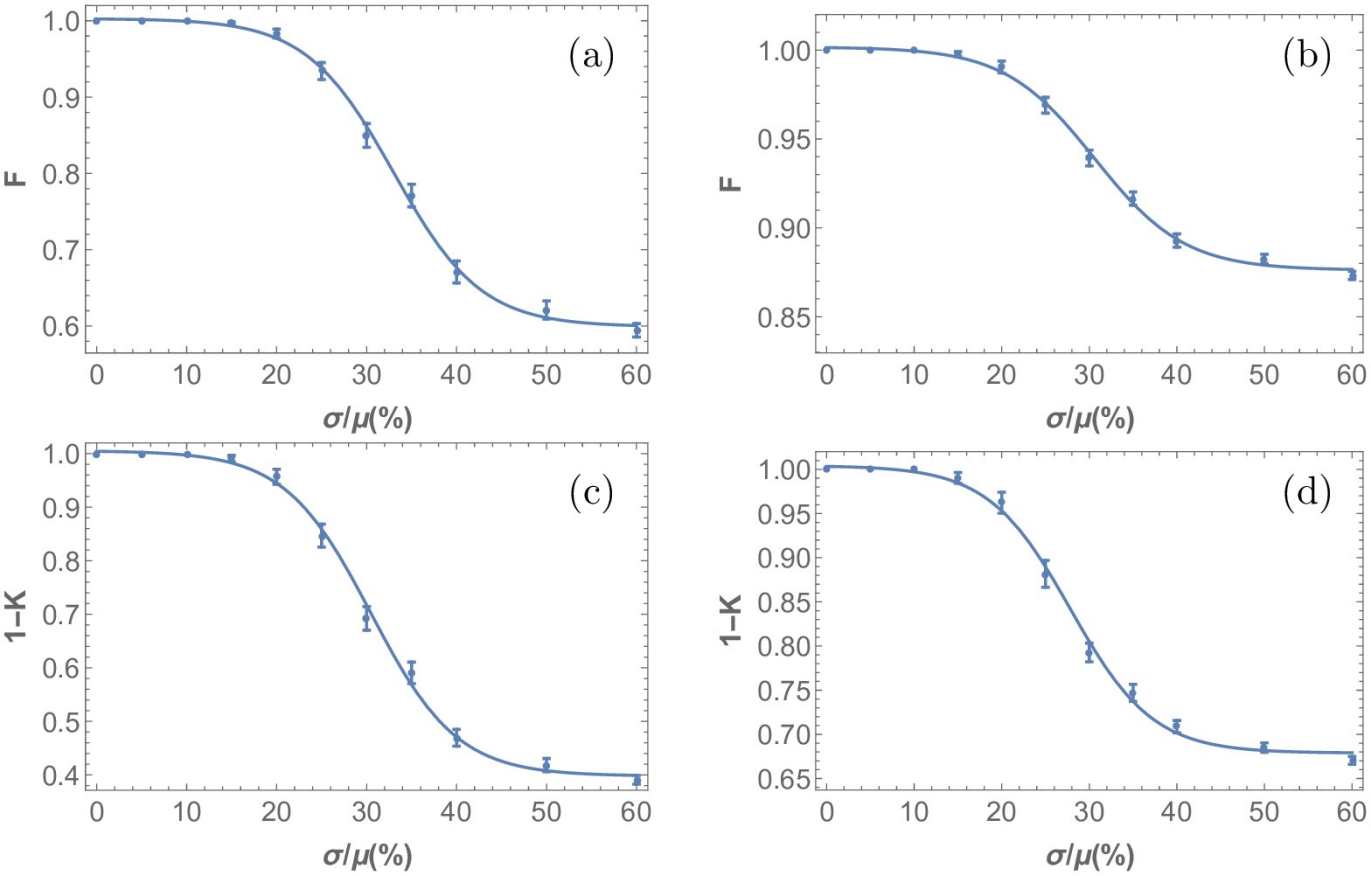
Probability distance measures applied to analyze response to stochasticity in R8 cell point pattern formation. (a)-(b) are generated using the fidelity, F, and (c)-(d) using the Kolmogorov distance, K, for nearest neighbor distances and angles respectively. Stochasticity was introduced in the model through drawing the parameter *D*_*u*_ and *D*_*s*_ in the differential equations Eqs 1–4 from a normal distribution with varying standard deviation, *σ*, and mean value, μ. The simulation results were compiled, and nearest R8 cell neighbor angle and distance distributions were obtained. Kolmogorov and fidelity of the resulting distributions where computed with reference to the perfect pattern case. Here, a value of 1 corresponds to a perfectly ordered pattern, and a value of 0 corresponds to a completely irregular pattern. This figure illustrates that the functional form is independent of the measure used.

Let us now discuss how this type of threshold behavior is also picked up by the more general measures of order given by Eqs 13 and 14. The results of applying these probability distance measures is shown in Fig 9. Note that saturation observed in the nearest angle distribution variance plot is apparent in both cases, and the responses of both observables (distances and angles) to stochasticity exhibit a universal sigmoidal functional form, implying threshold behavior. These measures reveal the same interesting quality as the variance: the pattern order exhibits an initial resistance to weak input stochasticity that eventually gives way to structural malformation at larger noise levels. The sigmoidal functional form confirms that the mechanism does exhibit robustness for *σ* < *σ*_*c*_. The fact that this is observed in the Kolmogorov measure, the fidelity, the bond orientation and variance measures supports the notion that such sigmoidal, threshold response to stochasticity is a universal feature (see supporting information, S4 Fig, for bond orientation sigmoidal response).

**Fig 10:**
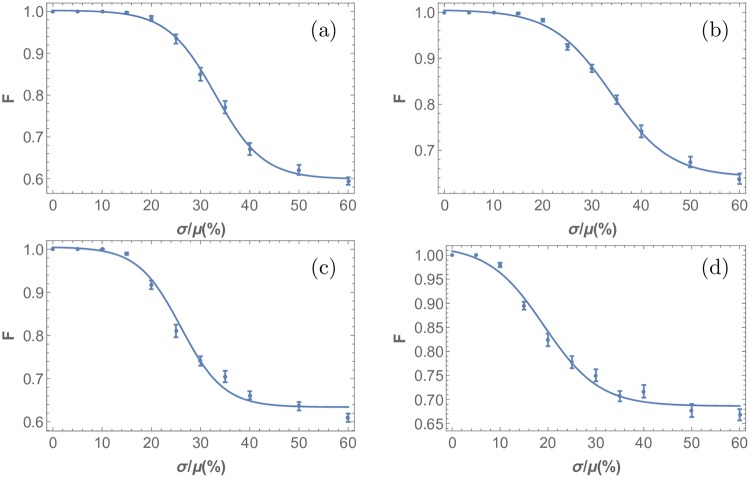
Universal threshold response to stochasticity in different model parameters. In each sub-plot, stochasticity is introduced in the following parameters of the underlying differential equations: (a) *D*_*u*_ and *D*_*s*_, (b) *P*_*a*_, (c) *S*, and (d) *G*. Introduction of parametric noise in all of the former parameters, produces a sigmoid response. The order measure used is the fidelity of the nearest neighbor distance probability distributions and sigmoid curves were used to fit the data.

Next, we turn to the question of universality with respect to how stochasticity is introduced. We observe that stochasticity in other model parameters also produces threshold behavior, indicating that the observed threshold response is a generic feature of this class of evolutionary models. Specifically, in Fig 10 we show the response to increasing stochasticity levels in the production rate of atonal *P*_*a*_, the production rate due to the activator *S*, and the production rate caused by the morphogenetic furrow *G*. In all these cases, there is also a regime *σ* < *σ*_*c*_ where the ordered pattern remains intact. For the threshold response in the case of noise in all parameters, except the ones determining *h*, the two remaining production rates *Pu* and *Ps* and for more cases see S2 Fig.

**Fig 11:**
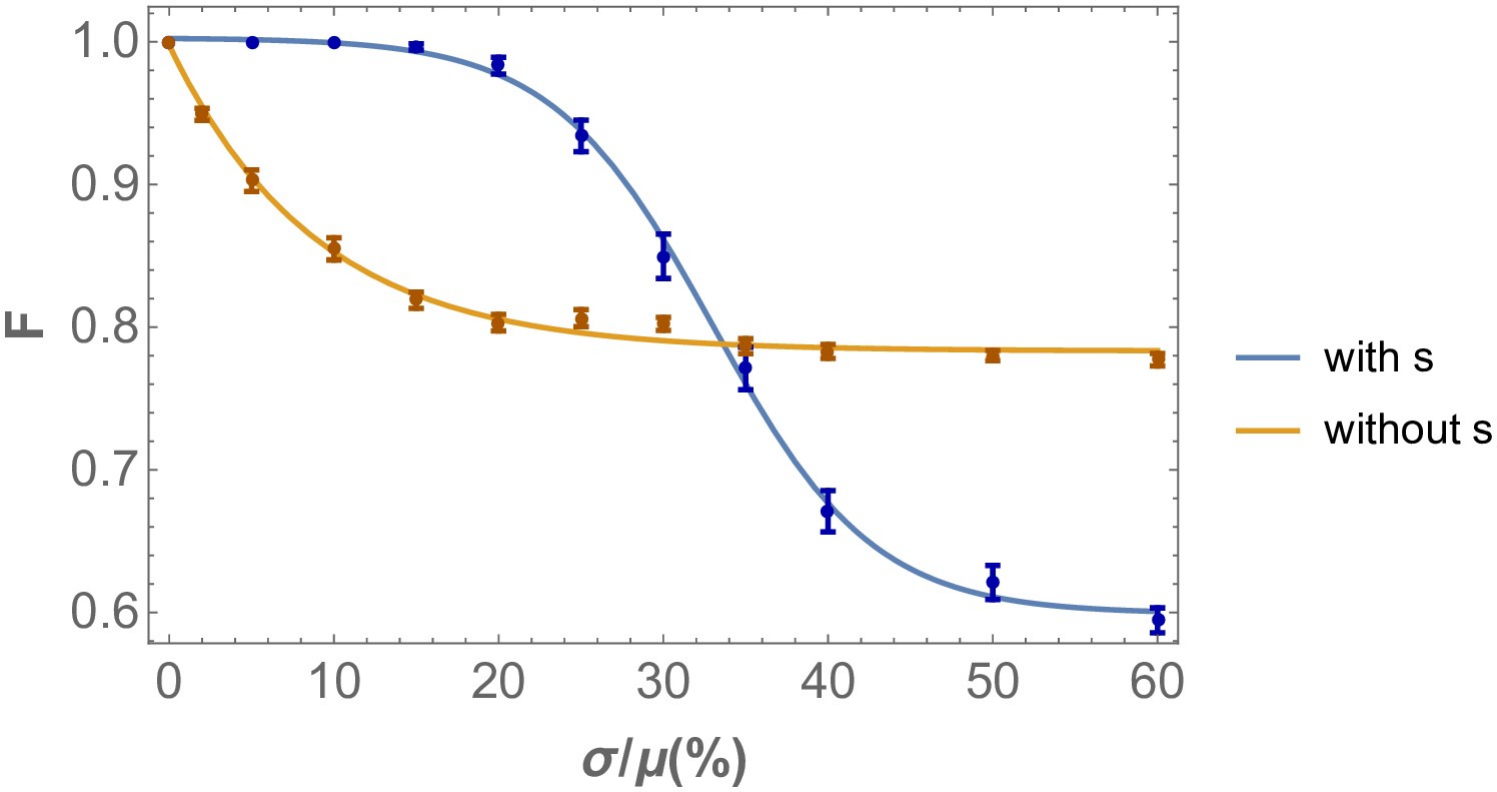
Robustness of Drosophila eye disc R8 cell pattern order versus stochasticity, with and without the diffusible activator Scabrous in the model. The analysis is applied in the same setting as Fig 9, i.e. stochasticity is introduced in the diffusivity of the morphogens *D*_*u*_ and *D*_*s*_. In the absence of the diffusible activator *s*, there is a much increased sensitivity to stochasticity for infinitesimal non-vanishing *σ*. The order measure used is the fidelity of the nearest neighbor distance probability distributions.

Next let us address the origin of the observed resilience of this model towards weak noise with *σ* < *σ*_*c*_, signified by the initial plateaus observed in Fig 10. The main reason for these wide regimes, exhibiting robustness with respect to parameter stochasticity, is the presence of Scabrous as a diffusible activator [7]. Introducing Scabrous decreases the sensitivity of the patterning mechanism to the shape of the inhibitory regions [7] visualized in Fig 2, and thus to slightly misplaced clusters, making the model more robust with respect to parametric perturbations. This effect can be identified in the ordered state where displaced cluster positions do not lead to dissipation of the order of the pattern, see S1 Fig. While this ingredient is not needed for the pattern to propagate, without this element even low stochasticity levels of *D*_*u*_ cause it to deteriorate, making the model of pattern formation biologically implausible [7]. This effect is quantitatively demonstrated in Fig 11, which compares the response to stochasticity with and without Scabrous as a diffusible activator. In the absence of Scabrous, with the same parameter set and with optimized initial conditions, there is an immediate deterioration of the patterned order upon introduction of infinitesimal noise. In contrast, the presence of Scabrous changes this functional relationship to the observed sigmoid response, thus introducing a resilience scale to the pattern formation mechanism set by the Scabrous production rate *P*_*s*_ and the activation threshold *s*_1_.

In addition to the robustness introduced by Scabrous, the threshold response of the order of the pattern to stochasticity in the various parameters shown in Fig 10 is a consequence of three further elements: (i) the spatial discreteness of the underlying lattice, (ii) the threshold activated response of cells to morphogen levels, and (iii) propagation of avalanches from anterior to posterior. As we now discuss, the robustness for low stochasticity levels is a consequence of the lattice discreteness and the threshold response of the cells to morphogen concentrations, whereas the sharp decay is a consequence of propagation of avalanches.

To understand how the discreteness of the underlying lattice contributes to robustness for low noise levels, consider the effect of altering the diffusivity of a morphogen. Since the local morphogen concentration drops off at distances on the order of a few cells, and the cells lie on a discrete hexagonal lattice, a small change in the diffusivity radius will not result in any difference unless it leads to the inclusion or exclusion of additional cells. A continuous change in the radius of inhibition will exclude or include new cells when it increases by a factor comparable to the lattice spacing, thus creating a threshold response. Alternatively, consider the effect of the morphogenetic furrow, mathematically denoted by Eq 4, which propagates with a constant speed through the lattice of cells. Two consecutive cells on along the direction of propagation of the morphogenetic furrow will receive the activation signal with a delay lag, *δt*. Only if the production rates, denoted by G in Eq 1, picked from the normal distributions differ above a finite threshold one will result to a misplaced cell. This effect is shown in the absence of *s* as a short range activator, when noise in introduced in G, in S5 Fig. An analytical approach to this effect and its contribution in error is carried out in [19].

Second, the threshold response of pattern formation to parameter stochasticity is linked to the threshold activation of cells in response to morphogen levels. The turn-on and turn-off of morphogens in the differential equations is governed by step functions with threshold values, *a*_*a*_, *a*_*u*_, *u*_1_, *h*_1_, *s*_1_. This has the consequence that the effect of morphogen concentration is binary, i.e. the parameter variation must be sufficient such that the concentration threshold is crossed for activation or inhibition to occur. Nevertheless, even for more continuous cell responses to morphogen levels, there is always a threshold value and the same argument can be made. To rule out whether the threshold response to stochasticity is an artifact of the binary functional form of the Heaviside functions appearing in Eqs 1–4, further simulations were performed, see S2 Appendix.

Finally, to explain the fast decay of the pattern beyond threshold stochasticity, the propagation of avalanches comes into play, an effect also identified by [7, 8]. Beyond *σ*_*c*_, R8 clusters start to get significantly elongated and misplaced. The first such elongated R8 cell cluster causes the next row of R8 cell clusters to be misplaced, leading to a cascade onset of pattern disorder. This avalanche effect is quantitatively discussed next, where we analyze the order of the pattern as a function of position.

### Increasing disorder of the pattern from posterior to anterior

**Fig 12:**
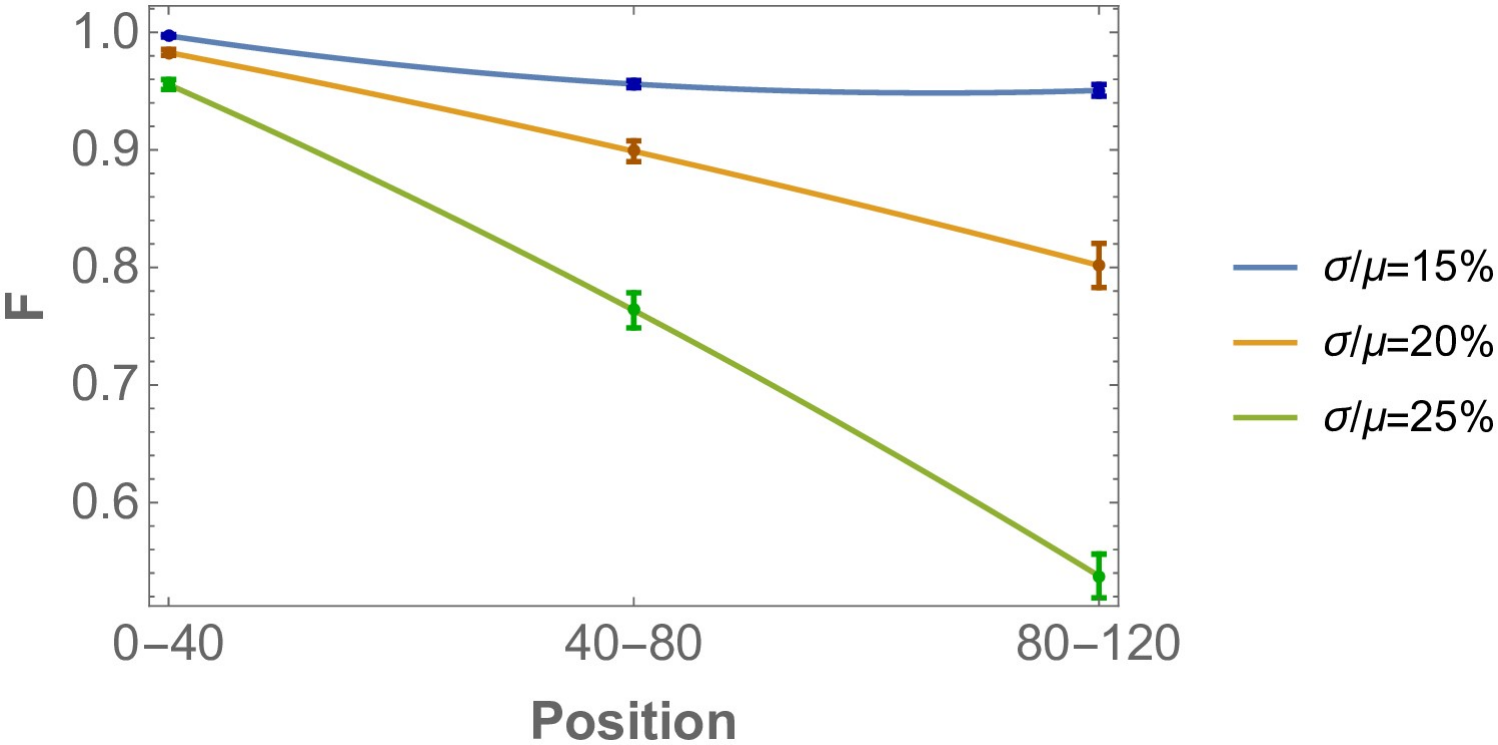
Decay of R8 pattern order as a function of position on the eye disc (same parameter set as in Fig 5). The local order measure is calculated as a function of position, from posterior to anterior. The x-axis refers to regions of the simulated eye disc 0-40, 40-80, 80-120 respectively, whereas the y-axis refers to the fidelity probability distance measure applied to nearest neighbor distance distributions. The three colored lines correspond to various stochasticity levels in *D*_*u*_ and *D*_*s*_, from *σ*/*μ* = 15% to *σ*/*μ* = 25% in steps of 5%. Local order generally decreases from posterior to anterior. Once the threshold of *σ*_*c*_/*μ* ≈ 20% is crossed, there is a very sharp spatial decay of the order of the pattern, signaling the underlying avalanche effect.

Here we use the fidelity measure to evaluate error propagation as a function of position on the spatial posterior-to-anterior axis. The mechanism of pattern formation relies delicately on carefully spaced precursor R8 cells that are placed in the posterior region before the growth process described by the differential equations is initiated. Their positions define the subsequent cluster spacing of activated R8 cells for the entire eye disc. In the presence of stochasticity, this information dissipates during the propagation of the morphogenetic furrow from posterior to anterior, since the ommatidial pattern on a column is defined by the shape of the inhibition signals emitted by the ommatidial row before it. It is thus expected that as the morphogenetic furrow travels from posterior to anterior, errors in the pattern order grow in an avalanche fashion. This effect is observed in the simulations, as shown in Fig 12, where the disorder of the pattern clearly increases from posterior to anterior.

Within the noise regime studied, the order parameter F decreases by two effects. First, the pattern becomes increasingly disordered as it propagates. Second, a larger part of the pattern becomes disordered. Interestingly, in the high noise regime, the pattern saturates to a value for F that does not increase further from posterior to anterior, see S6 Fig. The higher the noise, the faster it reaches to the saturated value of F. Thus, in the regime of high noise the disorder is largely due to a larger part of the pattern being in the disordered state. Conversely, for low noise regimes, the dominant effect is the intensification of disorder from posterior to anterior. These two effect are also universal, observed for all parameters, since they are the precursors of a threshold response to stochasticity.

**Fig 13:**
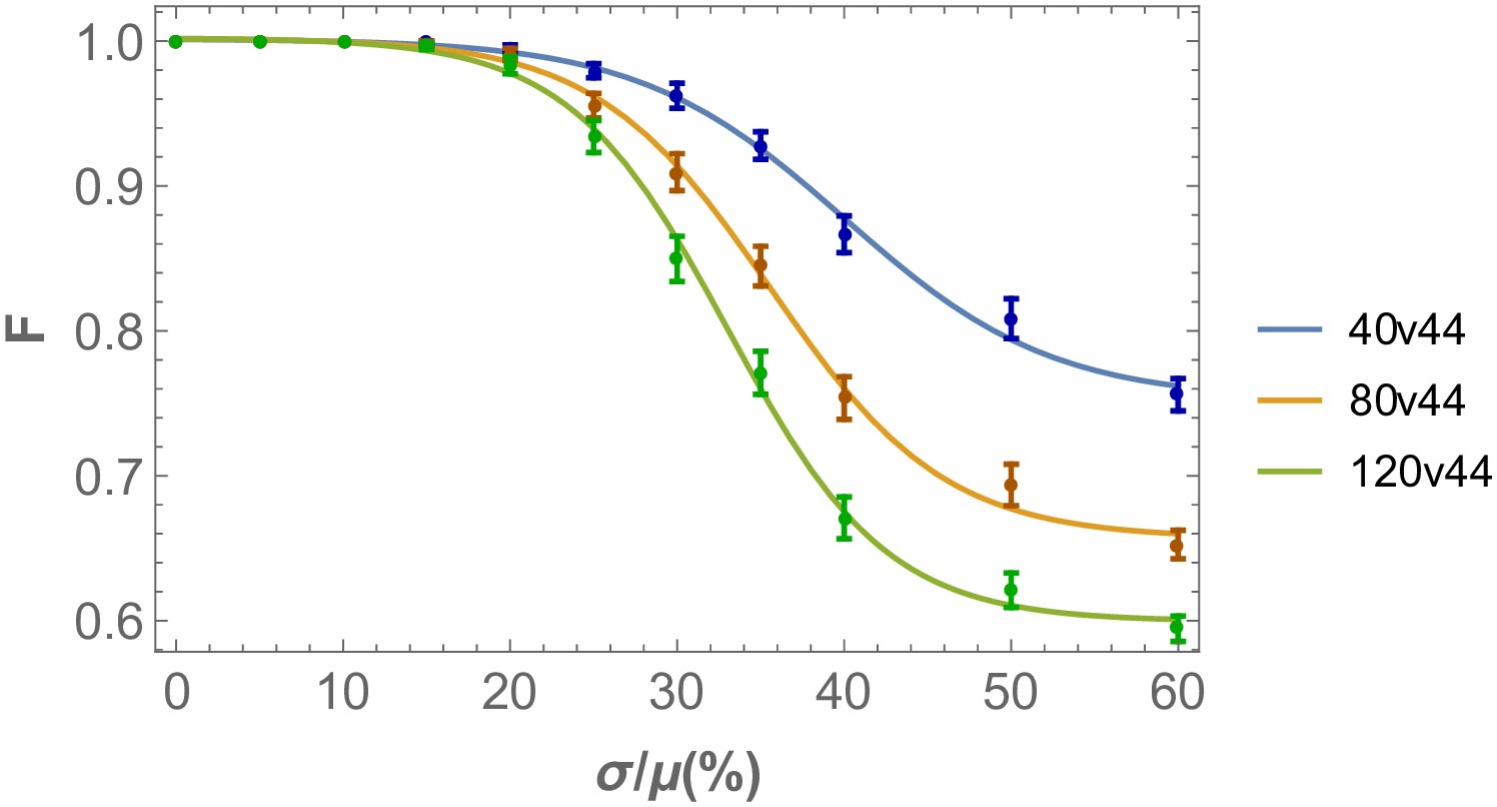
Pattern order as a function of eye disc size (same parameter sets as in Fig 8). The threshold response is more pronounced as the length of the eye disc in the direction of the morphogenetic furrow is increased. The order parameter is calculated for cell number 40, 80 and 120 while keeping the size perpendicular to the propagation direction of the morphogenetic furrow at 44 cells. The order measure used is the fidelity of the nearest neighbor distance probability distributions and noise is added on the diffusivity of the morphogens.

Previous studies have not, so far, quantitatively analyzed the consequences of finite system sizes on the order of the pattern. To illustrate these finite size effects on the threshold response quantitatively, in Fig 13 we plot the order parameter obtained from numerical simulations for different eye disc sizes, illustrating the effect of size on the robustness of the system. Here one clearly observes that the larger eye-disc, the sharper the threshold response to stochasticity. Since our numerical simulations were performed on lattices much smaller than the actual eye disc, the observed threshold effect can be extrapolated to be much more pronounced for realistic eye disc sizes.

This variation in the degree of dependency of the pattern order measure upon stochasticity levels across different eye disk sizes is analogous to the finite size scaling of order parameters commonly observed in interacting physical many body systems, such as Ising models on lattices. In analogy to phase transitions in the thermodynamic limit of such models, i.e. for infinite system sizes, here we observe precursor sigmoidal response to increasing stochasticity levels that becomes more pronounced for larger system (eye disk) sizes, ultimately culminating into a true phase transition as *L*_*x*_ → ∞. The structure of the underlying system of coupled dynamical equations suggests that this transition belongs to the universality class of directed percolation [32]. While a complete scaling analysis is beyond the scope of this study, it will be provided in a future publication.

## Conclusions

The model of coupled differential equations in Eqs 1–4, describing eye disc R8 cell differentiation, provides robust developmental mechanisms that lead to resilience against stochastic perturbations. This robustness can be attributed to the discrete nature of the underlying lattice and the threshold activation of cells to morphogens. In addition, the introduction of Scabrus, as rigorously illustrated in [7], leads to increased resilience against stochasticity, especially in stochasticity in the diffusivity of the inhibitor. Beyond a critical noise threshold *σ*_*c*_, there is an acute loss of order with increasing stochasticity due to avalanches of misplaced differentiated cells. This threshold value can be obtained by fitting the response with a sigmoid and is found to occur when the relative noise variation of a parameter reaches a certain value, distinguishing the work from more qualitative observations of robustness [7, 8]. Furthermore, the preliminary results of introducing noise to multiple parameters, see S2 Fig, suggest an additive effect of the respective variables on the system’s response to noise. This suggests that the number of parameters subject to fluctuations in the regulatory network change the quantitative threshold but the sigmoid response is conserved. Finally, when the R8 pattern formed in the absence of noise, this and previous studies [7] observe small variations from column to column in the pattern. In this analysis, we verified that these variations do not lead to loss of of order even for large system sizes and thus, strengthened the plausibility of this mathematical formulation.

This threshold response to noise is an essential characteristic of developmental systems. A lack of resilience would deem biological systems unable to produce complex, nearly perfect structures in an inherently noisy background. This is especially true in eye disc formation with large numbers of ∼ 800 ommatidia. The introduction of Scabrus creates a redundancy that supports the threshold response. The model system studied here exhibits biologically plausible robustness, identifying the sigmoid functional form as one of the emergent responses to noise of developmental models.

The expression of a gene depends on a probabilistic outcome determined by the upstream regulatory sequences that act in cis, and the binding and processivity of other molecules that act in trans. Thus, mutational changes in cis-regulatory elements and trans-regulatory factors can affect transcription levels and transcriptional noise [33]. These changes in transcriptional noise reveal some of the evolutionary constraints on cis-regulatory elements [34]. The sigmoid functional response buffers the system against perturbations. This functional form indicates the effect of Cryptic Genetic Variation (CGV). CGV is largely neutral until it is exposed in certain genetic backgrounds. Robustness in development allows for the accumulation of CGV without any observable changes in phenotype. Once the genetic background changes, for example through mutational perturbation, this previously-neutral bottled-up CGV can be released to produce strikingly different phenotypes [35].

Our analysis further illustrates that as the R8 clusters are specified, errors propagate, leading to increased irregularity from posterior to anterior. This rate is important as it suggests that there is an interplay between how large an eye disc is with how perfectly ordered it can be, with larger eye discs being more likely to accumulate some positional errors. This has interesting implications on limiting eye disc size, a hypothesis that can be investigated experimentally.

The structural order measures outlined in this paper can be used to quantify order of patterns in developmental systems containing local point patterns. The application of the radial pair correlation function for nearest neighbors, as specifically performed in this study, is useful for data sets that have non trivial periodic structure and are small in size. Specifically, the measure is useful for analysis of experimental data of the eye-disc, since the effect of curvature of the eye-disc is eliminated and restrictive boundary effects are resolved. The point-pattern order measure used here, based on probability distance, is independent of the functional form of the underlying nearest neighbor distributions and boundary conditions, thus making it applicable independent of the form of the point pattern.

## Supporting information

S1 Appendix Exact expression of the Laplace operator and noise. (PDF)

S2 Appendix Threshold response is not an artifact of the Heaviside functions. (PDF)

S1 Table Numerical parameters of complete mathematical model. This parameter set is taken from previous studies, plugging it in the mathematical equations produces the desired pattern in a biologically plausible regime [7].
(PNG)

S1 Fig Quantification of order in the R8 cell pattern in the absence of noise as a function of anterior to posterior. In this plot, the fidelity order measure for nearest neighbor distances is applied on an R8 cell pattern generated by the mathematical model. The mathematical model is evaluated on an elongated cell grid of 200 vs 44 cells in the case of no noise with the parameters in S1 Table. The order measure used is the fidelity of the nearest neighbor distance distributions. In this case, the fidelity order measure uses as a reference the pattern of the first 0-40 cells in the direction of the morphogenetic furrow (chosen as the x-direction in this paper) and uses as a target the pattern of the 40-80, 80-120 and 160-200 cells as shown in the x-axis of the plot. The first data point starts at 1 and then saturates to a value of 9.85 for the rest of the slices. The plot supports the claim that, in the zero noise case, the resulting pattern retains its degree of order as it propagates. The reason that the first data point is higher than the other is that the first column of cells is placed manually as an initial condition and it is placed slightly to the right to avoid the effects of reflective boundary conditions. Details on the quantitative measure of order can be found in the Methods section of the main text.
(PDF)

S2 Fig Parametric variation leads to a threshold response when it is applied to a variety of parameters and in different combinations. (a) Parametric variation is applied in equal degree for all parameters except the ones that determine *h* propagation. (b) Parametric variation is applied in variable amplitude for the pair *P*_*a*_ and *D*_*u*_, *D*_*s*_. (c) Parametric variation on *P*_*u*_. (d) Parametric variation on *P*_*s*_. (e) Parametric variation in equal degree for all the production rates and *D*_*u*_, *D*_*s*_ as in [7]. The order measure used is the fidelity of the nearest neighbor distance distributions. Parametric variation was not applied on the elements that define *h* as it is approximated analytically and the pattern exhibited great sensitivity to such variations.
(PDF)

S3 Fig Probability distance order measures lead to threshold response when uniform distribution is used as a reference. (a)-(b) are generated using the fidelity, F, and (c)-(d) using the Kolmogorov distance, K, for nearest neighbor distances and angles respectively. The probability distance measures are applied on the R8 point pattern with noise added in the model for the parameters *D*_*u*_ and *D*_*s*_. The probability distance order measures need a reference distribution to quantify order. In contrast to the rest of the plots in this article, the reference distributions where chosen to be the uniform distributions. The threshold response to stochasticity is apparent in the plots. As the pattern deteriorates with increasing noise, it approaches the uniform distribution. Thus, the fidelity between the distributions increases and the Kolomogorov distance decreases.
(PDF)

S4 Fig Sigmoid response of bond orientation order parameter. The bond orientation order parameter also exhibits the sigmoid response to noise. This further supports the fact that the response of the system to noise is physical. To calculate the bond orientation order, the Voronoi diagram method was used to precisely determine the nearest neighbors in the point pattern.
(PDF)

S5 Fig The threshold response is apparent, although at a lower noise level, even in the absence of *s* as a local inhibitor. (PDF)

S6 Fig Fidelity order measure as a function of position on the simulated eye-disc. In this plot, noise was introduced in Du and Ds. Similarly to Fig 12, the plot shows the order of the pattern as a function of position from anterior to posterior. The x-axis refers to regions of the simulated eye disc 0-40, 40-80, 80-120 respectively, whereas the y-axis refers to the fidelity probability distance measure applied to nearest neighbor distance distributions. The conclusion is that the pattern saturates to a value of F. As the noise is increased, this saturation happens earlier in the eye disc.
(PDF)
