# Supplementary material for: Threshold response to stochasticity in morphogenesis
Source: PLoS One. 2019 Jan 30;14(1):e0210088. doi: 10.1371/journal.pone.0210088 (PMC6353092; doi:10.1371/journal.pone.0210088)
Supplement: S1 Appendix — (PDF) [file pone.0210088.s001.pdf]

S1 Appendix. Exact expression of the Laplace operator and noise.

The discretized form of the Laplace operator for the diffusivity of u in the hexagonal lattice arrangement of cells is given by: $D_u \Delta u^i = \sum_{<ij>} D_u^{ij}(u_i - u_j)$. In this equation, D_u^{ij} denotes the diffusivity across cell wall i and cell wall j, u_i denotes the concentration of u in the cell with cell wall i and $< ij >$ denotes all six nearest neighbors, rigorously defined by via a Voronoi diagram of the hexagonal lattice. In addition, the diffusion of a morphogen from cell wall i to cell wall j is equal to the diffusion from cell wall j to cell wall i, realized by the mathematical relationship $D_u^{ij} = D_u^{ji}$. When there is noise, each D_u^{ij} is picked from a gaussian distribution as explained in the subsection Introducing Noise. Only in the case where there is no noise, we get the simplification that all the diffusivities are the same, $D_u^{ij} = D_u$, equal to the reference value given in S1 Table. Similarly, for the discretized form of the Laplace operator for the diffusivity of s, the following mathematical relation was used: $D_s \Delta s^i = \sum_{<ij>} D_s^{ij}(s_i - s_j)$. However, since the variation in diffusivity across cell boundaries is morphogen independent, the following relation is applied: $D_s^{ij} = \frac{D_s}{D_u} D_u^{ij}$. In this model, the propagation of h is approximated analytically and its diffusivity was kept constant.
