# Supplementary material for: Threshold response to stochasticity in morphogenesis
Source: PLoS One. 2019 Jan 30;14(1):e0210088. doi: 10.1371/journal.pone.0210088 (PMC6353092; doi:10.1371/journal.pone.0210088)
Supplement: S2 Appendix — (PDF) [file pone.0210088.s002.pdf]

S2 Appendix. Threshold response is not an artifact of the Heaviside functions. The mathematical model defined by Eqs 1-4 includes eight infinitely sharp Heaviside functions. The Heaviside functions were placed as an approximation to the more realistic sigmoid curves. However, with the current parameter set, the pattern does not propagate when one directly replaces them. Since the interest of this study is to use the verified parameter sets in S1 Table, the Heaviside functions were replaced with ramp functions. The ramp functions did allow the pattern to propagate in the desired parameter space, with the minor adjustment of increasing the production of the short ranged activator s by a factor of 1.95. The ramp functions where defined as:

$$\begin{cases} R(x) = 0 & x \leq (1-p)x_0 \\ R(x) = \frac{1}{2x_0 p}(x - x_0(1-p)) & x_0(1-p) < x < x_0(1+p) \\ R(x) = 1 & x \geq (1+p)x_0 \end{cases} \tag{1}$$

Where x_0 the threshold value that was used for the Heaviside functions and 2p is the percentage of the value that the gradual response occurs. All the Heaviside functions were replaced except the ones for h propagation, where great sensitivity was identified. Applying the order measures are plotted in the figure bellow. The system retains a threshold response to noise.

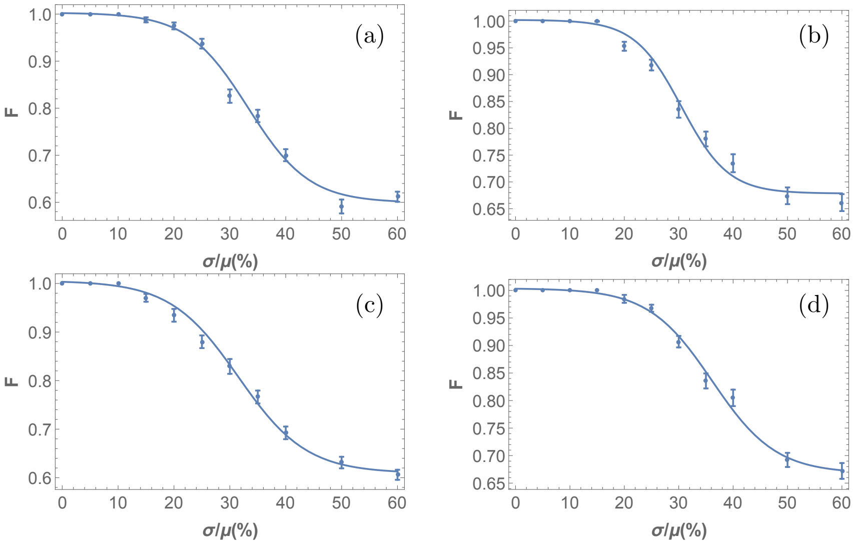

S2 Appendix Fig. The threshold response is apparent, although at a lower noise level, even when the Heaviside functions are replaced with smoother ramp functions. (a)-(b) are performed for ramp functions with $p = 0.1$ and in (c)-(d) $p = 0.2$. In (a),(c) parametric variation is applied on both the diffusivities of u and s and in (b),(d) for the production rate Pa.
