# Supplementary figures and images for: Threshold response to stochasticity in morphogenesis
Source: PLoS One. 2019 Jan 30;14(1):e0210088. doi: 10.1371/journal.pone.0210088 (PMC6353092; doi:10.1371/journal.pone.0210088)

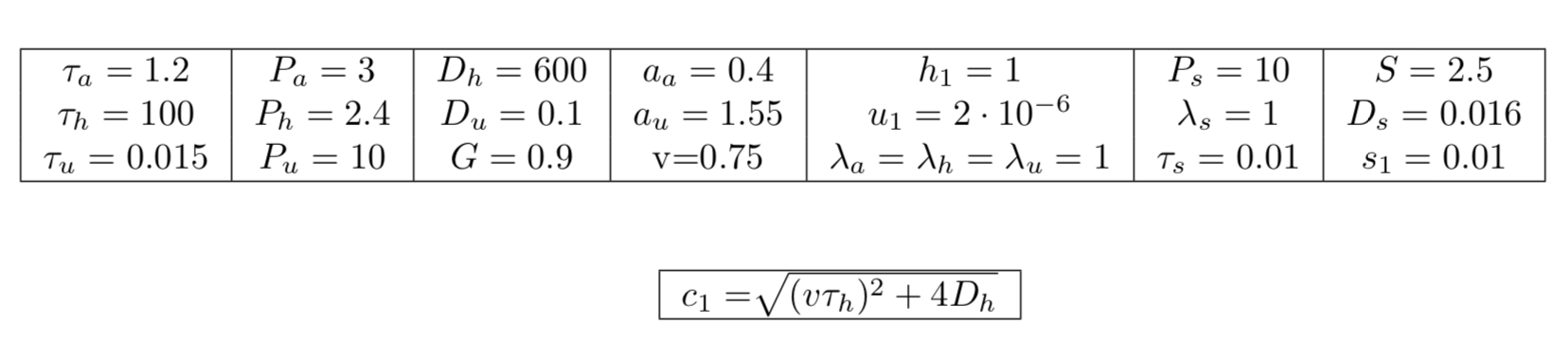

Supplement: S1 Table — This parameter set is taken from previous studies, plugging it in the mathematical equations produces the desired pattern in a biologically plausible regime [7]. (PNG) [file pone.0210088.s003.png]

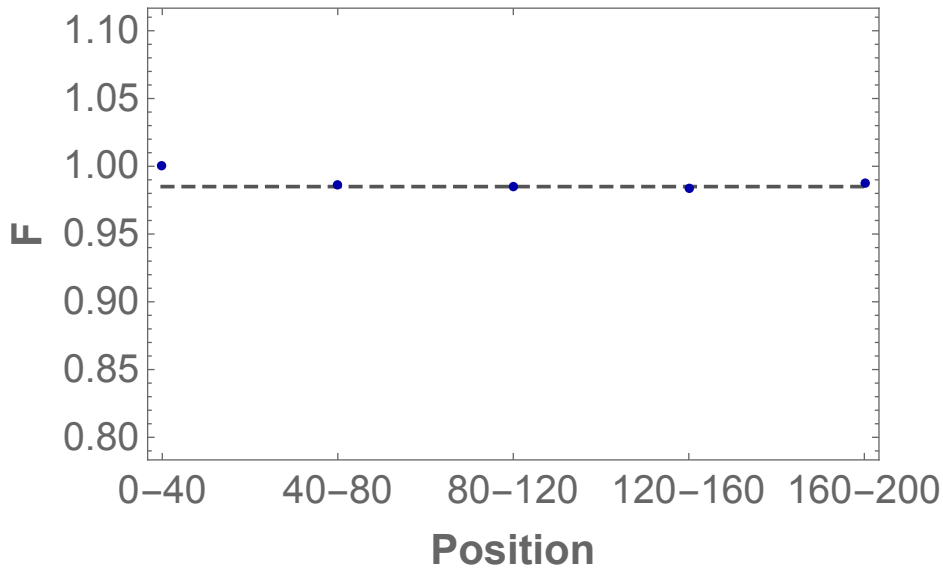

Supplement: S1 Fig — In this plot, the fidelity order measure for nearest neighbor distances is applied on an R8 cell pattern generated by the mathematical model. The mathematical model is evaluated on an elongated cell grid of 200 vs 44 cells in the case of no noise with the parameters in S1 Table. The order measure used is the fidelity of the nearest neighbor distance distributions. In this case, the fidelity order measure uses as a reference the pattern of the first 0-40 cells in the direction of the morphogenetic furrow (chosen as the x-direction in this paper) and uses as a target the pattern of the 40-80, 80-120 and 160-200 cells as shown in the x-axis of the plot. The first data point starts at 1 and then saturates to a value of 9.85 for the rest of the slices. The plot supports the claim that, in the zero noise case, the resulting pattern retains its degree of order as it propagates. The reason that the first data point is higher than the other is that the first column of cells is placed manually as an initial condition and it is placed slightly to the right to avoid the effects of reflective boundary conditions. Details on the quantitative measure of order can be found in the Methods section of the main text. (PDF) [file pone.0210088.s004.pdf]

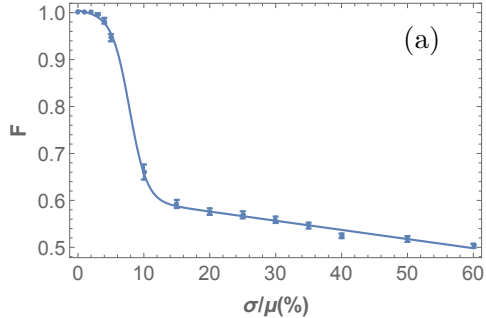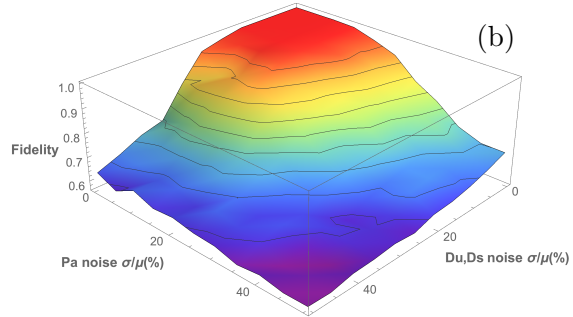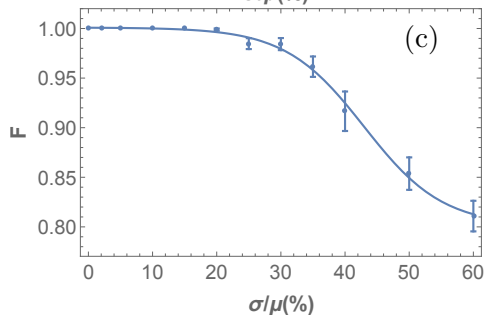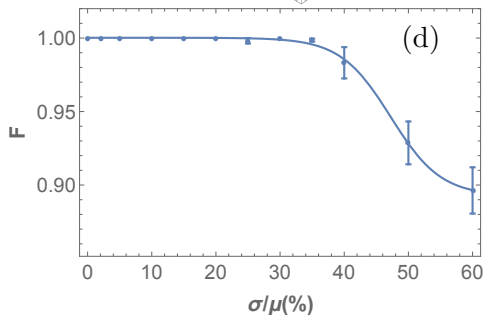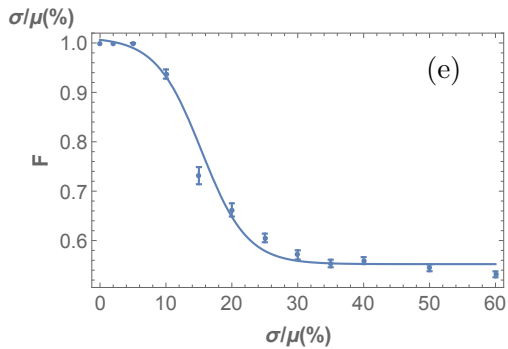

Supplement: S2 Fig — (a) Parametric variation is applied in equal degree for all parameters except the ones that determine h propagation. (b) Parametric variation is applied in variable amplitude for the pair Pa and Du, Ds. (c) Parametric variation on Pu. (d) Parametric variation on Ps. (e) Parametric variation in equal degree for all the production rates and Du, Ds as in [7]. The order measure used is the fidelity of the nearest neighbor distance distributions. Parametric variation was not applied on the elements that define h as it is approximated analytically and the pattern exhibited great sensitivity to such variations. (PDF) [file pone.0210088.s005.pdf]

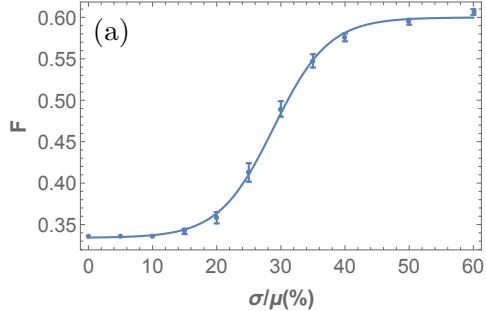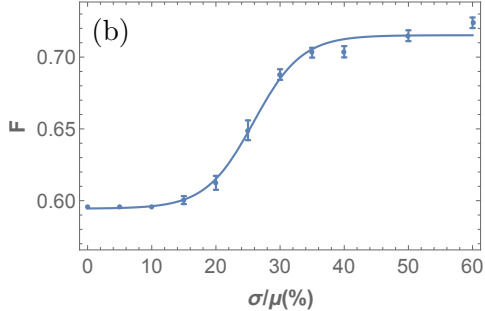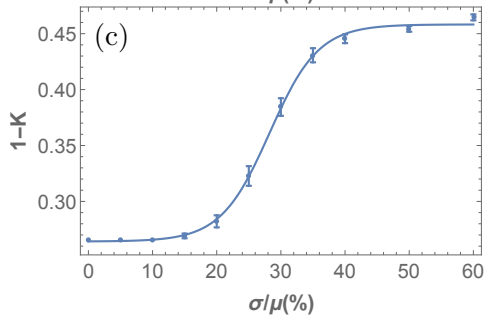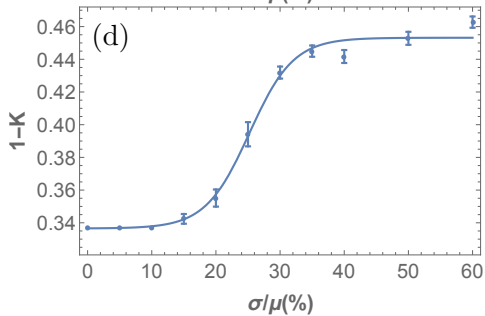

Supplement: S3 Fig — (a)-(b) are generated using the fidelity, F, and (c)-(d) using the Kolmogorov distance, K, for nearest neighbor distances and angles respectively. The probability distance measures are applied on the R8 point pattern with noise added in the model for the parameters Du and Ds. The probability distance order measures need a reference distribution to quantify order. In contrast to the rest of the plots in this article, the reference distributions where chosen to be the uniform distributions. The threshold response to stochasticity is apparent in the plots. As the pattern deteriorates with increasing noise, it approaches the uniform distribution. Thus, the fidelity between the distributions increases and the Kolomogorov distance decreases. (PDF) [file pone.0210088.s006.pdf]

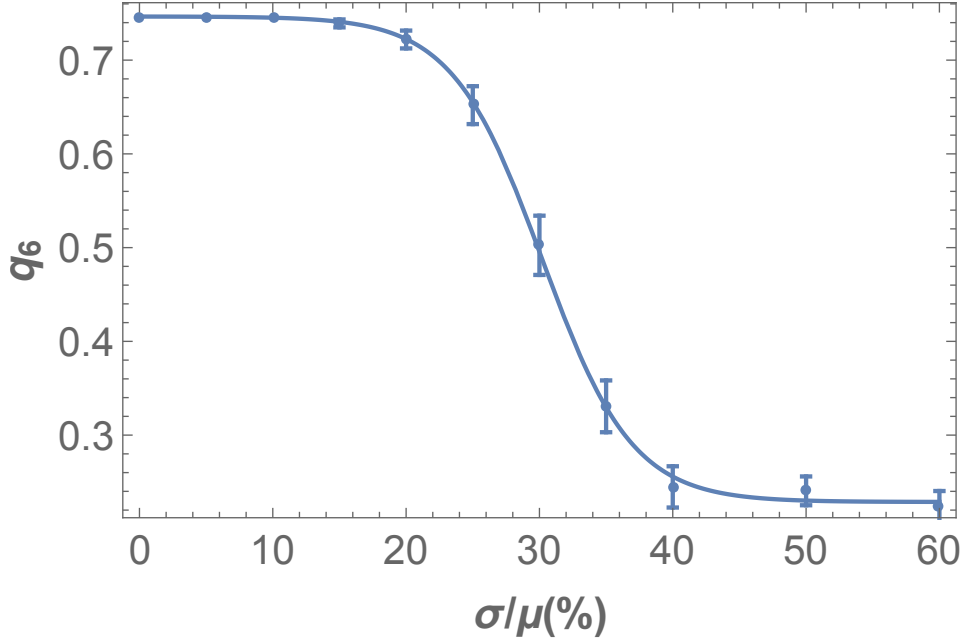

Supplement: S4 Fig — The bond orientation order parameter also exhibits the sigmoid response to noise. This further supports the fact that the response of the system to noise is physical. To calculate the bond orientation order, the Voronoi diagram method was used to precisely determine the nearest neighbors in the point pattern. (PDF) [file pone.0210088.s007.pdf]

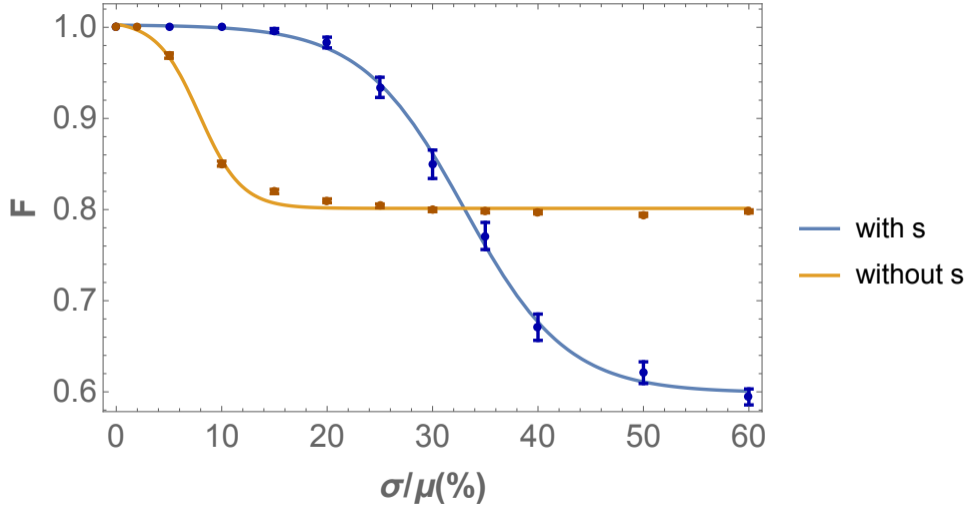

Supplement: S5 Fig — (PDF) [file pone.0210088.s008.pdf]

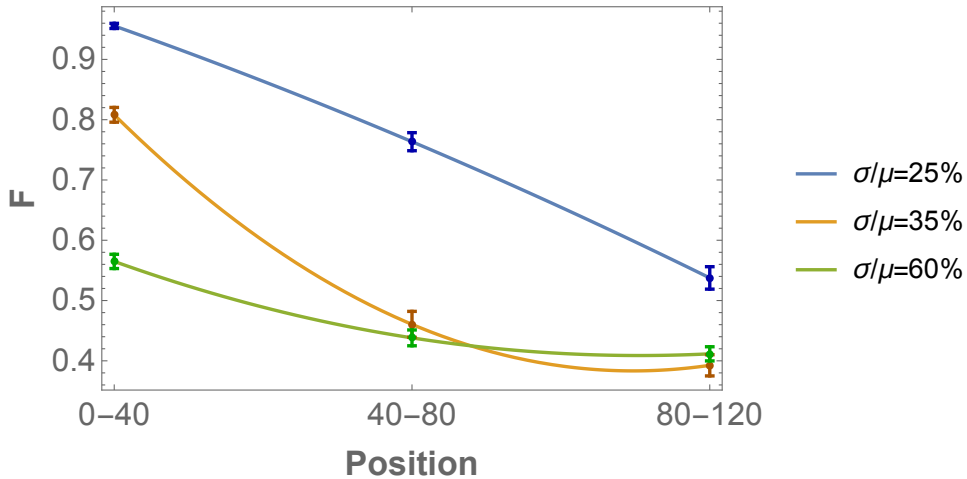

Supplement: S6 Fig — In this plot, noise was introduced in Du and Ds. Similarly to Fig 12, the plot shows the order of the pattern as a function of position from anterior to posterior. The x-axis refers to regions of the simulated eye disc 0-40, 40-80, 80-120 respectively, whereas the y-axis refers to the fidelity probability distance measure applied to nearest neighbor distance distributions. The conclusion is that the pattern saturates to a value of F. As the noise is increased, this saturation happens earlier in the eye disc. (PDF) [file pone.0210088.s009.pdf]
